# Three-Dimensional Simulations of Anisotropic Slip Microflows Using the Discrete Unified Gas Kinetic Scheme

**DOI:** 10.3390/e24070907

**Published:** 2022-06-30

**Authors:** Wenqiang Guo, Guoxiang Hou

**Affiliations:** School of Naval Architecture and Ocean Engineering, Huazhong University of Science and Technology, Wuhan 430074, China; gwq@hust.edu.cn

**Keywords:** anisotropic slip, boundary condition, DUGKS, superhydrophobic surface, oscillating wall motion

## Abstract

The specific objective of the present work study is to propose an anisotropic slip boundary condition for three-dimensional (3D) simulations with adjustable streamwise and spanwise slip length by the discrete unified gas kinetic scheme (DUGKS). The present boundary condition is proposed based on the assumption of nonlinear velocity profiles near the wall instead of linear velocity profiles in a unidirectional steady flow. Moreover, a 3D corner boundary condition is introduced to the DUGKS to reduce the singularities. Numerical tests validate the effectiveness of the present method, which is more accurate than the bounce-back and specular reflection slip boundary condition in the lattice Boltzmann method. It is of significance to study the lid-driven cavity flow due to its applications and its capability in exhibiting important phenomena. Then, the present work explores, for the first time, the effects of anisotropic slip on the two-sided orthogonal oscillating micro-lid-driven cavity flow by adopting the present method. This work will generate fresh insight into the effects of anisotropic slip on the 3D flow in a two-sided orthogonal oscillating micro-lid-driven cavity. Some findings are obtained: The oscillating velocity of the wall has a weaker influence on the normal velocity component than on the tangential velocity component. In most cases, large slip length has a more significant influence on velocity profiles than small slip length. Compared with pure slip in both top and bottom walls, anisotropic slip on the top wall has a greater influence on flow, increasing the 3D mixing of flow. In short, the influence of slip on the flow field depends not only on slip length but also on the relative direction of the wall motion and the slip velocity. The findings can help in better understanding the anisotropic slip effect on the unsteady microflow and the design of microdevices.

## 1. Introduction

Surface characteristics play a critical role in designing and fabricating microfluidic devices. Superhydrophobicity is an important aspect of surface characteristics, which can significantly control flow and reduce drag [1,2,3,4,5,6,7,8,9,10]. Based on the knowledge of fluid mechanics, the no-slip boundary condition is valid at the solid–liquid interface. However, the liquid slip velocity is observed on the superhydrophobic surface. Unlike gas slip caused by Knudsen effects, liquid slip is modelled with two strategies [11]: introducing the force that repels water in the multiphase system; and modelling the slip boundary condition. For the former strategy, the forces are not well understood and determined. Existing research recognizes the critical role played by the latter strategy; therefore, it is emphasized in the present work.

The appropriate boundary condition is an important area in the simulation of fluid flows [12,13]. Recently, researchers have shown an increased interest in slip boundary conditions. For example, Min and Kim [14,15] directly modelled the hydrophobic wall with Navier’s slip boundary condition by direct numerical simulation (DNS) based on the macroscopic Navier–Stokes equations. However, the Navier’s slip boundary condition cannot be introduced to the lattice-Boltzmann-based methods, because the lattice-Boltzmann-based methods track the particle distribution function instead of the macroscopic variables. The lattice Boltzmann method (LBM) is appropriate for simulating mesoscopic physics that are hard to describe macroscopically. It has the advantages of simple algorithms, natural parallelization, and saving computing costs for simulating microflow [16], which proves to be a promising tool. Slip boundary conditions have been proposed and employed in the LBM, such as bounce-back and specular reflection (BSR) [11,17], discrete full diffusive and specular reflection (DSR) [18,19], discrete full diffusive and bounce-back (DBB) [20,21], and tangential momentum accommodation coefficient (TMAC) scheme [22]. Coupled with Navier’s slip model [23], liquid slip can be characterized and adjusted by slip length. With assumption of the 2D unidirectional flow at a lower Reynolds number, the relation between the slip length and combination parameter of the coupled schemes [24,25,26] is determined. However, it is not valid in three-dimensional (3D) flows and turbulent flows. Moreover, the slip should be considered in streamwise and spanwise directions [1]. Existing studies about rice leaves have found that the anisotropic groove-like microstructures can lead to the anisotropic slip behavior of water droplets on the surfaces [27,28,29]. More recent studies have considered superhydrophobic surfaces with randomly distributed textures in streamwise and spanwise directions [8,30,31,32,33,34,35], indicating that the slip is anisotropic. For example, both spanwise and streamwise slip lengths have been measured on a randomly textured superhydrophobic surface [35]. Therefore, the present study considers the anisotropic slip in streamwise and spanwise directions for the 3D system based on the nonlinear velocity profiles near the wall, which is close to the actual situation.

Moreover, Guo et al. proposed a new finite-volume scheme named discrete unified gas kinetic scheme (DUGKS) based on the lattice Boltzmann equation [36]. The DUGKS has also been proved to be a promising numerical tool to simulate fluid flow [37,38,39,40,41,42], such as 3D turbulent channel flow. It is found that the DUGKS, even with a coarse nonuniform mesh, is overall better than the LBM [37]. So, the numerical simulation will be performed by the DUGKS in this work. Up to now, no attention has been paid to the 3D slip boundary condition in the DUGKS. Therefore, in the present study, the new slip boundary condition is proposed for the DUGKS instead of the LBM. The DUGKS is performed to simulate 3D flows with the proposed slip boundary condition. It is hoped that we provide a superior method to describe and characterize anisotropic slip on superhydrophobic surfaces.

To study the effect of anisotropic slip on flows in benchmark geometries, the present method is applied to the lid-driven cavity flow. It is an important problem in the field of fluid mechanics due to its applications, such as cooling of electronic gadgets, oil extraction, design of heat exchangers, solar ponds, acoustic liner, float glass productions, insulation materials, multiscreen gadgets for nuclear reactors, coating, food processing, crystal growth, etc. [43,44,45,46,47,48,49]. Moreover, it has capability in exhibiting important phenomena such as eddies, secondary flows, instabilities, transition, and turbulence [50]. The liquid slip flow in two-sided, orthogonal, oscillating, micro-lid-driven cavities has largely been oversighted. Recently, two-sided motion [51,52,53,54] and oscillatory flows in the cavity have also caught the necessary attention, except single-sided steady flows. The purposes of the literature cornering oscillatory flows in lid-driven cavities include: 

1. To test and validate numerical solvers, such as least-squares finite element methods [55], Taylor-series-expansion- and least-squares-based lattice Boltzmann methods [56] and conservative level-set methods [57]. 2. To understand industrial applications, such as surface viscometer [58] and optimization of fluid mixing [59,60]. 3. To understand the flow characteristics, such as the single-sided oscillatory rarefied gas flows inside two- and three-dimensional cavities [61,62,63], and two-sided oscillating flows in two-dimensional lid-driven cavities [64].

The studies mentioned above have been solely restricted to the no-slip flow; the liquid slip flow in lid-driven cavities have largely been oversighted. Slip should be carefully considered in the design of micro-devices with moving parts. This work will generate fresh insight into the effects of anisotropic slip on the 3D flow in a two-sided oscillating micro-lid-driven cavity. In this work, for the first time, there are mainly two types of slip distribution: (1) pure streamwise slip emerges on both top and bottom wall surfaces; (2) both streamwise and spanwise slips emerge on the top wall surface. In a 3D global coordinate system, the unit velocity vector of the moving top and bottom walls is (1,0,0) and (0,1,0), respectively. The motion direction of the top and bottom walls is orthogonal. To the best of the authors’ knowledge, no such study has been conducted before.

It is hoped that the present study will provide a superior method and contribute to a deeper understanding of the anisotropic slip. The paper is organized as follows. In Section 2, the D3Q19 lattice model and DUGKS are introduced, and the new slip boundary condition and corner boundary condition for the D3Q19 lattice model are derived theoretically. In Section 3, numerical validation is performed by simulating the 3D microchannel flow. Numerical results of 3D flow in a two-sided, oscillating, lid-driven cavity are discussed in Section 4. Section 5 gives the conclusions.

## 2. Numerical Methods

### 2.1. D3Q19 Lattice Model

**Figure 1:**
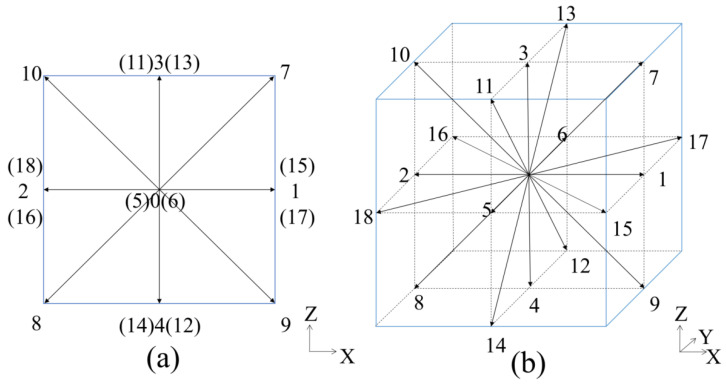
D3Q19 lattice model (**a**) 2D view; (**b**) 3D view.

D3Q19 lattice model [65] is adopted in this work. As shown in Figure 1, there are 19 discrete velocities in the D3Q19 lattice model, including one rest velocity (*α* = 0) and 18 non-rest velocities (*α* = 1, ..., 18).

As shown in Table 1, the velocity set includes the velocities $\left\{\xi_{\alpha }\right\}$ and the corresponding weights $\left\{W_{\alpha }\right\}$. The speed of the sound is $c=\sqrt{3RT}=1$.

### 2.2. Discrete Unified gas Kinetic Scheme

The discrete Boltzmann equation with the Bhatnagar–Gross–Krook (BGK) collision model [66] is the governing equation of the DUGKS:

(1) $$\frac{\partial f_{\alpha }}{\partial t}+\xi_{\alpha }\cdot \nabla f_{\alpha }=\Omega \equiv \frac{f_{\alpha }{}^{eq}-f_{\alpha }}{\tau }$$

It is assumed that fluid particles move with the velocity $\xi_{\alpha }$ at position x and time *t*, so the velocity distribution function is $f_{\alpha }=f_{\alpha }\left(x,\xi_{\alpha },t\right)$. Ω and *τ* represent the collision term and relaxation time, respectively.

$f_{\alpha }{}^{eq}$ represents the Maxwellian equilibrium distribution function, which is approximated by Taylor expansion around zero particle velocity at low Mach number:

(2) $$f_{\alpha }{}^{eq}=W_{\alpha }\rho \left[1+\frac{\xi_{\alpha }\cdot u}{c_{s}^{2}}+\frac{{\left(\xi_{\alpha }\cdot u\right)}^{2}}{2c_{s}^{4}}-\frac{{\left|u\right|}^{2}}{2c_{s}^{2}}\right],\;c_{s}^{2}=RT$$

The velocities $\left\{\xi_{\alpha }\right\}$ and the corresponding weights $\left\{W_{\alpha }\right\}$ are presented in Table 1.

The computational domain is divided into cuboid cells *V_i,j,k_* = Δ*x_i_*Δ*y_j_*Δ*z_k_* centered at **x***_i,j,k_* = (*x_i_*, *y_j_*, *z_k_*) in the DUGKS. As a new finite volume scheme, the volumes-averaged values of the distribution function and collision term need to be computed. For example, the volumes-averaged value of the distribution function $f_{\alpha }^{n}(x_{i,j,k})$ is computed as,

(3) $$f_{\alpha }^{n}(x_{i,j,k})=\frac{1}{\left|V_{i,j,k}\right|}\int_{V_{i,j,k}}f_{\alpha }\left(x,t^{n}\right)dV$$

In the DUGKS, the governing equation needs to be integrated on each cell, and the time step Δ*t* is assumed to be constant. Equation (1) is integrated on a cell *V_i,j,k_* centered at **x***_i,j,k_* from time $t^{n}=n\Delta t$ to time $t^{n+1}=\left(n+1\right)\Delta t$, and the evolution of the distribution is advanced from *t^n^* to *t^n+^*^1^ as,

(4) $$f_{\alpha }^{n+1}-f_{\alpha }^{n}=-\frac{\Delta t}{\left|V_{i,j,k}\right|}{ℱ}_{\alpha }^{n+1/2}+\frac{\Delta t}{2}\left[\Omega_{\alpha }^{n}+\Omega_{\alpha }^{n+1}\right]$$

The scalar variable ${ℱ}_{\alpha }^{n+1/2}$ represents the flux across the cell interface,

(5) $$\begin{array}{l} {ℱ}_{\alpha }^{n+1/2}(x_{i,j,k})=\int_{\partial V_{i,j,k}}\left(\xi_{\alpha }\cdot n\right)f_{\alpha }^{n+1/2}(x_{b})dS=\\ =[f_{\alpha }^{n+1/2}(x_{i,j,k}+0.5\Delta x_{i}e_{x})-f_{\alpha }^{n+1/2}(x_{i,j,k}-0.5\Delta x_{i}e_{x})]\xi_{\alpha ,x}\Delta y_{j}\Delta z_{k}\\ +[f_{\alpha }^{n+1/2}(x_{i,j,k}+0.5\Delta y_{j}e_{y})-f_{\alpha }^{n+1/2}(x_{i,j,k}-0.5\Delta y_{j}e_{y})]\xi_{\alpha ,y}\Delta x_{i}\Delta z_{k}\\ +[f_{\alpha }^{n+1/2}(x_{i,j,k}+0.5\Delta z_{k}e_{z})-f_{\alpha }^{n+1/2}(x_{i,j,k}-0.5\Delta z_{k}e_{z})]\xi_{\alpha ,z}\Delta x_{i}\Delta y_{j} \end{array}$$

where $f_{\alpha }^{n+1/2}(x_{b})$ represents the distribution at the cell interface **x***_b_* at the time *t*^n+1/2^ = *t*^n^ + *h* (*h* = Δ*t*/2), and **e**_x_, **e**_y_, and **e**_z_ are unit vectors in x, y, and z directions, respectively.

For clarity, new distribution functions are introduced:

(6) $${\tilde{f}}_{\alpha }^{n}\equiv f_{\alpha }^{n}-\frac{\Delta t}{2}\Omega \left(f_{\alpha }^{n}\right),{\tilde{f}}_{\alpha }^{+,n}\equiv f_{\alpha }^{n}+\frac{\Delta t}{2}\Omega \left(f_{\alpha }^{n}\right)$$

The collision term can be expanded in the BGK collision model, and Equation (6) can be converted to the following equations:

(7) $$f_{\alpha ,j}^{n}=\frac{2\tau }{2\tau +\Delta t}{\tilde{f}}_{\alpha ,j}^{n}+\frac{\Delta t}{2\tau +\Delta t}f_{\alpha ,j}^{eq,n}\;,\;{\tilde{f}}_{\alpha ,j}^{+,n}=\frac{2\tau -\Delta t}{2\tau +\Delta t}{\tilde{f}}_{\alpha ,j}^{n}+\frac{2\Delta t}{2\tau +\Delta t}f_{\alpha ,j}^{eq,n}\;.$$

The evolution equation of DUGKS from *t^n^* to *t^n+^*^1^ is simplified as:

(8) $${\tilde{f}}_{\alpha ,j}^{n+1}={\tilde{f}}_{\alpha ,j}^{+,n}-\frac{\Delta t}{\left|V_{i,j,k}\right|}{ℱ}_{\alpha ,j}^{n+1/2}$$

Based on the conservation of mass, momentum, the density *ρ,* and velocity **u** can be computed from ${\tilde{f}}_{\alpha }$:

(9) $$\rho^{n}=\sum_{\alpha }{\tilde{f}}_{\alpha }^{n},\rho^{n}u^{n}=\sum_{\alpha }\xi_{\alpha }{\tilde{f}}_{\alpha }^{n}$$

All other forms of the distribution function can be expressed in terms of ${\tilde{f}}_{\alpha }$ and $f_{\alpha }{}^{eq}$. So, the distribution function ${\tilde{f}}_{\alpha }$ is mainly computed in the code.

The critical step is to evaluate the interface flux ${ℱ}_{\alpha ,j}^{n+1/2}$. The midpoint integral formula is employed to evaluate ${ℱ}_{\alpha ,j}^{n+1/2}$, due to its easy implementation and fast computation. For DUGKS with higher-order accuracy, more intermediate time steps need to be selected; for example, the flux at the cell interface at t * = *t*_n_ + ∆*t*/6 and t * = *t*_n_+ 3∆*t*/4 need calculating.

In the present study, Equation (1) is integrated within a half time step (*h* = Δ*t*/2) along the characteristic line with the endpoint (**x***_b_*) located at the cell interface, and the following formula is obtained:

(10) $$f_{\alpha }^{n+1/2}\left(x_{b}\right)-f_{\alpha }^{n}\left(x_{b}-h\xi_{\alpha }\right)=\frac{h}{2}\left[\Omega \left(f_{\alpha }^{n+1/2}\left(x_{b}\right)\right)+\Omega \left(f_{\alpha }^{n}\left(x_{b}-h\xi_{\alpha }\right)\right)\right]$$

Similarly, new distribution functions are introduced and can be computed by expanding the collision term:

(11) $${\bar{f}}_{\alpha }^{n+1/2}\left(x_{b}\right)\equiv f_{\alpha }^{n+1/2}\left(x_{b}\right)-\frac{h}{2}\Omega \left(f_{\alpha }^{n+1/2}\left(x_{b}\right)\right)=\frac{2\tau +h}{2\tau }f_{\alpha }^{n+1/2}\left(x_{b}\right)-f_{\alpha }^{eq,n+1/2}\left(x_{b}\right)$$

(12) $$\begin{array}{c} {\bar{f}}_{\alpha }^{+,n}\left(x_{b}-h\xi_{\alpha }\right)\equiv f_{\alpha }^{n}\left(x_{b}-h\xi_{\alpha }\right)+\frac{h}{2}\Omega \left(f_{\alpha }^{n}\left(x_{b}-h\xi_{\alpha }\right)\right),\\ {\bar{f}}_{\alpha }^{+,n}=\frac{2\tau -h}{2\tau +h}{\bar{f}}_{\alpha }^{n}+\frac{2h}{2\tau +h}f_{\alpha }^{eq,n}. \end{array}$$

With new distribution functions, Equation (10) is converted to the following equation:

(13) $${\bar{f}}_{\alpha }^{n+1/2}\left(x_{b}\right)={\bar{f}}_{\alpha }^{+,n}\left(x_{b}-h\xi_{\alpha }\right)$$

With the Taylor expansion around the endpoint (**x***_b_*) located at the cell interface, the right term of Equation (13) can be approximated as:

(14) $${\bar{f}}_{\alpha }^{+,n}\left(x_{b}-h\xi_{\alpha }\right)={\bar{f}}_{\alpha }^{+,n}\left(x_{b}\right)-h\xi_{\alpha }\cdot \nabla {\bar{f}}_{\alpha }^{+,n}\left(x_{b}\right)$$

where ${\bar{f}}_{\alpha }^{+,n}\left(x_{b}\right)$ and its gradients $\nabla {\bar{f}}_{\alpha }^{+,n}\left(x_{b}\right)$ can be approximated by the linear interpolations. In x-direction, e.g.,

(15) $$\begin{array}{l} \frac{\partial {\bar{f}}_{\alpha }^{+,n}\left(x_{i,j,k}+0.5\Delta x_{i}e_{x}\right)}{\partial x}\approx \frac{{\bar{f}}_{\alpha }^{+,n}\left(x_{i+1,j,k}\right)-{\bar{f}}_{\alpha }^{+,n}\left(x_{i,j,k}\right)}{(\Delta x_{i}+\Delta x_{i+1})/2},\\ {\bar{f}}_{\alpha }^{+,n}\left(x_{i,j,k}+0.5\Delta x_{i}e_{x}\right)\approx {\bar{f}}_{\alpha }^{+,n}\left(x_{i,j,k}\right)+\frac{\partial {\bar{f}}_{\alpha }^{+,n}\left(x_{i,j,k}+0.5\Delta x_{i}e_{x}\right)}{\partial x}\frac{\Delta x_{i}}{2}, \end{array}$$

The distribution function ${\bar{f}}_{\alpha }^{+,n}$ can be computed from ${\tilde{f}}_{\alpha }$, as follows,

(16) $${\bar{f}}_{\alpha }^{+,n}=\frac{2\tau -h}{2\tau +\Delta t}{\tilde{f}}_{\alpha }^{n}+\frac{3h}{2\tau +\Delta t}f_{\alpha }^{eq,n}$$

Then, we obtain the function ${\bar{f}}_{\alpha }^{n+1/2}\left(x_{b}\right)$. The density and velocity at the cell interface can also be evaluated, which can be used for the equilibrium distribution function $f_{\alpha }^{eq,n+1/2}\left(x_{b}\right)\;$,

(17) $${\rho^{n+1/2}|}_{x_{b}}=\sum_{\alpha }{\bar{f}}_{\alpha }^{n+1/2}\left(x_{b}\right),{{\left(\rho u\right)}^{{}^{n+1/2}}|}_{x_{b}}=\sum_{\alpha }\xi_{\alpha }{\bar{f}}_{\alpha }^{n+1/2}\left(x_{b}\right)$$

Finally, the flux ${ℱ}_{\alpha ,j}^{n+1/2}$ is evaluated according to Equation (5) after the distribution function $f_{\alpha }^{n+1/2}$ at the cell interface is determined by Equation (11). The tracked distribution function ${\tilde{f}}_{\alpha }$ can be updated to the next time step after the flux is obtained.

Particularly, the following equation will be used in the DUGKS,

(18) $${\tilde{f}}_{\alpha }^{+,n}=\frac{4}{3}{\bar{f}}_{\alpha }^{+,n}-\frac{1}{3}{\tilde{f}}_{\alpha }^{n}$$

For the present DUGKS, the relaxation time *τ* is computed from *τ = μ/p*, where *μ* is the dynamic viscosity coefficient. *p* (*p = ρRT*) is the pressure, where *R* is the specific gas constant. In the following simulations, the temperature *T* is constant in the isothermal flow with $c_{s}{}^{2}=RT=1/3$. The time step Δ*t* is determined by the Courant–Friedrichs–Lewy (CFL) condition [67], which is independent of the relaxation time *τ* for all flow regimes.

### 2.3. The Present Slip Boundary Condition

It can be seen intuitively that, considering the actual case with anisotropic slip, slip boundary conditions derived by adopting two-dimensional unidirectional flow are not valid. Therefore, a new anisotropic slip boundary condition is proposed in 3D flows. In this work, the anisotropic slip boundary condition is characterized and constructed by adjusting the relative magnitude of the streamwise and spanwise slip lengths. It is noted that *x*, *y,* and *z* denote the streamwise, spanwise, and wall-normal directions, respectively. It is noted that the DUGKS tracks the distribution function ${\tilde{f}}_{\alpha }$, unlike the LBM.

Considering the impermeable wall boundary (*U*_W*z*_ = 0), the unknown distributions are obtained by the specular reflection (${\tilde{f}}_{\alpha }^{sr}$) and the stress exerted by the wall (${\tilde{f}}_{\alpha }^{w}$).

(19) $${\tilde{f}}_{\alpha }={\tilde{f}}_{\alpha }^{sr}+{\tilde{f}}_{\alpha }^{w}(\xi_{\alpha }\cdot n>0)$$

**Figure 2:**
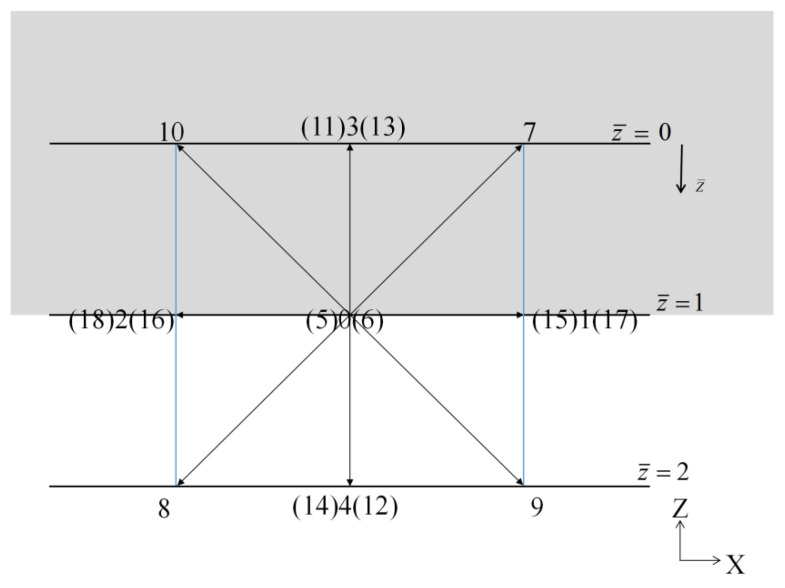
2D sketch of the upper horizontal wall boundary in 3D.

As shown in Figure 2, the unknown distributions are ${\tilde{f}}_{4},{\tilde{f}}_{8},{\tilde{f}}_{9},{\tilde{f}}_{12},{\tilde{f}}_{14}$.

The specular reflection ${\tilde{f}}_{\alpha }^{sr}$ can be obtained by:

(20) $${\tilde{f}}_{4}^{sr}={\tilde{f}}_{3},{\tilde{f}}_{8}^{sr}={\tilde{f}}_{10},{\tilde{f}}_{9}^{sr}={\tilde{f}}_{7},{\tilde{f}}_{12}^{sr}={\tilde{f}}_{13},{\tilde{f}}_{14}^{sr}={\tilde{f}}_{11}$$

With ${\tilde{f}}_{\alpha }^{sr}$ determined, $U_{SR}$ can be obtained by:

(21) $$\rho =\sum_{\alpha }{\tilde{f}}_{\alpha }$$

(22) $$\rho U_{SR}=\sum_{\xi_{\alpha }\cdot n\leq 0}{\left({\tilde{f}}_{\alpha }\xi_{\alpha }\right)}_{\left|\right|}+\sum_{\xi_{\alpha }\cdot n>0}{\left({\tilde{f}}_{\alpha }^{sr}\xi_{\alpha }\right)}_{\left|\right|}$$

The stress ${\tilde{f}}_{\alpha }^{w}$ contributes to the change in tangential momentum caused by the wall surface, as shown in the following equations:

(23) $$\sigma^{\prime }\rho \left(U_{W}-U_{SR}\right)=\sum_{\xi_{\alpha }\cdot n>0}{\left({\tilde{f}}_{\alpha }^{w}\xi_{\alpha }\right)}_{\left|\right|}$$

(24) $$0=\sum_{\xi_{\alpha }\cdot n>0}{\left({\tilde{f}}_{\alpha }^{w}\xi_{\alpha }\right)}_{⊥}$$

(25) $$0={\tilde{f}}_{\alpha }^{w}(\mathrm{for}\;\mathrm{normal}\;\mathrm{direction})$$

$U_{W}$ and $U_{SR}$ are the tangential velocity of the wall and the average tangential velocity under the specular reflection by the impermeable boundary, respectively. $\sigma^{\prime }$ represents a modified tangential momentum accommodation coefficient. n is the normal direction of the wall toward the fluid field. The subscripts “||” and “⊥” represent the tangential and normal directions, respectively. The sum of normal parts is zero, ensuring that the function ${\tilde{f}}_{\alpha }^{w}$ only changes the tangential momentum. It is also shown that the calculation of density is not determined by $\sum {\tilde{f}}_{\alpha }^{w}$.

The following relations can be obtained for the case in Figure 2.

(26) $$x\;\text{-}\;\mathrm{direction}:\;\rho U_{SRx}={\tilde{f}}_{1}-{\tilde{f}}_{2}+2({\tilde{f}}_{7}-{\tilde{f}}_{10})$$

(27) $$y\;\text{-}\;\mathrm{direction}:\;\rho U_{SRy}={\tilde{f}}_{8}-{\tilde{f}}_{5}+2({\tilde{f}}_{13}-{\tilde{f}}_{11})$$

The momentum can change due to the shear stress imposed on the wall:

(28) $$x\;\text{-}\;\mathrm{direction}:{\sigma^{\prime }}_{x}\rho \left(U_{Wx}-U_{SRx}\right)=\sum_{\xi_{\alpha }\cdot n>0}{\left({\tilde{f}}_{\alpha }^{w}\xi_{\alpha }\right)}_{x}={\tilde{f}}_{9}^{w}-{\tilde{f}}_{8}^{w}$$

(29) $$y\;\text{-}\;\mathrm{direction}:{\sigma^{\prime }}_{y}\rho \left(U_{Wy}-U_{SRy}\right)=\sum_{\xi_{\alpha }\cdot n>0}{\left({\tilde{f}}_{\alpha }^{w}\xi_{\alpha }\right)}_{y}={\tilde{f}}_{12}^{w}-{\tilde{f}}_{14}^{w}$$

For positive normal direction of the wall (i.e., +z direction):

(30) $$0=\sum_{\xi_{\alpha }\cdot n>0}{\left({\tilde{f}}_{\alpha }^{w}\xi_{\alpha }\right)}_{z}={\tilde{f}}_{4}^{w}+{\tilde{f}}_{8}^{w}+{\tilde{f}}_{9}^{w}+{\tilde{f}}_{12}^{w}+{\tilde{f}}_{14}^{w}$$

(31) $$0={\tilde{f}}_{4}^{w}$$

Based on the Maxwell equilibrium distribution function and Ref. [22], the density can be calculated,

(32) $$\rho ={\tilde{f}}_{0}+{\tilde{f}}_{1}+{\tilde{f}}_{2}+{\tilde{f}}_{5}+{\tilde{f}}_{6}+{\tilde{f}}_{15}+{\tilde{f}}_{16}+{\tilde{f}}_{17}+{\tilde{f}}_{18}+2({\tilde{f}}_{3}+{\tilde{f}}_{7}+{\tilde{f}}_{10}+{\tilde{f}}_{11}+{\tilde{f}}_{13})$$

Then, ${\tilde{f}}_{8}^{w},\;{\tilde{f}}_{9}^{w},\;{\tilde{f}}_{12}^{w},\;{\tilde{f}}_{14}^{w}$ and $U_{SRx},\;U_{SRy}$ are still unknown. ${\sigma^{\prime }}_{x},{\sigma^{\prime }}_{y}$ are the manually adjustable parameters, which are related to the streamwise and spanwise slip lengths, respectively.

There are five known equations in the system:

(33) $$\{\begin{array}{c} \begin{array}{c} \rho U_{SRx}={\tilde{f}}_{1}-{\tilde{f}}_{2}+2({\tilde{f}}_{7}-{\tilde{f}}_{10})\\ \rho U_{SRy}={\tilde{f}}_{8}-{\tilde{f}}_{5}+2({\tilde{f}}_{13}-{\tilde{f}}_{11})\\ {\sigma^{\prime }}_{x}\rho \left(U_{Wx}-U_{SRx}\right)=\sum_{\xi_{\alpha }\cdot n>0}{\left({\tilde{f}}_{i}^{w}\xi_{\alpha }\right)}_{x}={\tilde{f}}_{9}^{w}-{\tilde{f}}_{8}^{w}\\ {\sigma^{\prime }}_{y}\rho \left(U_{Wy}-U_{SRy}\right)=\sum_{\xi_{\alpha }\cdot n>0}{\left({\tilde{f}}_{i}^{w}\xi_{\alpha }\right)}_{y}={\tilde{f}}_{12}^{w}-{\tilde{f}}_{14}^{w} \end{array}\\ 0={\tilde{f}}_{8}^{w}+{\tilde{f}}_{9}^{w}+{\tilde{f}}_{12}^{w}+{\tilde{f}}_{14}^{w} \end{array}$$

To make the system closed, the hypothesis is proposed:

(34) $${\tilde{f}}_{8}^{w}=-{\tilde{f}}_{9}^{w}\;or\;{\tilde{f}}_{12}^{w}=-{\tilde{f}}_{14}^{w}$$

In summary, a new slip boundary condition is proposed for the upper horizontal wall boundary in 3D:

(35) $$\begin{array}{l} {\tilde{f}}_{4}={\tilde{f}}_{3}\\ {\tilde{f}}_{8}={\tilde{f}}_{10}-\frac{1}{2}{\sigma^{\prime }}_{x}\left\{\rho U_{Wx}-({\tilde{f}}_{1}-{\tilde{f}}_{2}+2({\tilde{f}}_{7}-{\tilde{f}}_{10}))\right\}\\ {\tilde{f}}_{9}={\tilde{f}}_{7}+\frac{1}{2}{\sigma^{\prime }}_{x}\left\{\rho U_{Wx}-({\tilde{f}}_{1}-{\tilde{f}}_{2}+2({\tilde{f}}_{7}-{\tilde{f}}_{10}))\right\}\\ {\tilde{f}}_{12}={\tilde{f}}_{13}+\frac{1}{2}{\sigma^{\prime }}_{y}\left\{\rho U_{Wy}-({\tilde{f}}_{6}-{\tilde{f}}_{5}+2({\tilde{f}}_{13}-{\tilde{f}}_{11}))\right\}\\ {\tilde{f}}_{14}={\tilde{f}}_{11}-\frac{1}{2}{\sigma^{\prime }}_{y}\left\{\rho U_{Wy}-({\tilde{f}}_{6}-{\tilde{f}}_{5}+2({\tilde{f}}_{13}-{\tilde{f}}_{11}))\right\} \end{array}$$

With the external forcing term, the local velocities u are computed by,

(36) $$u=\frac{1}{\rho }\sum_{i}\xi_{\alpha }{\tilde{f}}_{\alpha }+\frac{\Delta t\cdot \vec{a}}{2}$$

To eliminate the numerical slip due to force tangential to the wall, the external forcing term is introduced to the new slip boundary condition:

(37) $$\begin{array}{l} {\tilde{f}}_{4}={\tilde{f}}_{3}\\ {\tilde{f}}_{8}={\tilde{f}}_{10}-\frac{1}{2}{\sigma^{\prime }}_{x}\left\{\rho (U_{Wx}-0.5\Delta ta_{x})-({\tilde{f}}_{1}-{\tilde{f}}_{2}+2({\tilde{f}}_{7}-{\tilde{f}}_{10}))\right\}\\ {\tilde{f}}_{9}={\tilde{f}}_{7}+\frac{1}{2}{\sigma^{\prime }}_{x}\left\{\rho (U_{Wx}-0.5\Delta ta_{x})-({\tilde{f}}_{1}-{\tilde{f}}_{2}+2({\tilde{f}}_{7}-{\tilde{f}}_{10}))\right\}\\ {\tilde{f}}_{12}={\tilde{f}}_{13}+\frac{1}{2}{\sigma^{\prime }}_{y}\left\{\rho (U_{Wy}-0.5\Delta ta_{y})-({\tilde{f}}_{6}-{\tilde{f}}_{5}+2({\tilde{f}}_{13}-{\tilde{f}}_{11}))\right\}\\ {\tilde{f}}_{14}={\tilde{f}}_{11}-\frac{1}{2}{\sigma^{\prime }}_{y}\left\{\rho (U_{Wy}-0.5\Delta ta_{y})-({\tilde{f}}_{6}-{\tilde{f}}_{5}+2({\tilde{f}}_{13}-{\tilde{f}}_{11}))\right\} \end{array}$$

Similar manipulations can be applied to the lower wall and side walls boundary.

### 2.4. Relation between the Combination Parameters and Slip Lengths

Then, the relation between combination parameters (${\sigma^{\prime }}_{x},{\sigma^{\prime }}_{y}$) and slip lengths (*b_x_*, *b_y_*) is deduced to implement the new slip boundary condition. Previous research on the derivation of the relation is studied by taking the two-dimensional unidirectional steady flow as an example, which is expressed as [24,25,26]:

(38) $$\rho =const,u_{y}=0,a_{y}=0,\frac{\partial \phi }{\partial x}=0,\frac{\partial \phi }{\partial t}=0$$

where ϕ denotes flow variable, such as the velocity or density.

In this work, it is assumed that the anisotropic slip includes two components in streamwise and spanwise directions. Take the liquid slip on a horizontal plane (perpendicular to the *z*-axis) as an example. The slip length includes two components, *b_x_*, *b_y_* in the x and y directions, respectively. With the assumption of anisotropic slip, the simulation and characterization of the slip effect on a superhydrophobic surface can match the actual situation.

The upper wall in 3D is employed to derive the relationship between the parameters ${\sigma^{\prime }}_{x},{\sigma^{\prime }}_{y}$ and the slip lengths *b_x_*, *b_y_*.

The flow near the wall satisfies the continuity equation:

(39) $$\frac{\partial \rho }{\partial t}+\frac{\partial \rho u_{x}}{\partial x}+\frac{\partial \rho u_{y}}{\partial y}+\frac{\partial \rho u_{z}}{\partial z}=0$$

With the assumption of no density change in the incompressible flow, the continuity equation can be written as,

(40) $$\frac{\partial u_{x}}{\partial x}+\frac{\partial u_{y}}{\partial y}+\frac{\partial u_{z}}{\partial z}=0$$

In the 3D directional flows, the velocity distribution near the wall is assumed to be satisfied the following equations:

(41) $$\frac{\partial u_{x}}{\partial y}=0,\;\;\frac{\partial u_{y}}{\partial x}=0,\;\;u_{z}=0$$

As shown in Figure 2, the local coordinate system $\left(\bar{x},\bar{y},\bar{z}\right)$ in 3D is established in the lattice unit, where a node on the wall is served as the origin. The $\bar{x}-\bar{y}$ plane is located on the wall. The $\bar{z}$ direction is normal along the wall. In the local coordinate system, the boundary of the upper wall is located at the plane $\bar{z}=1$, and the solid is located in the region ($\bar{z}<1$), e.g., the plane $\bar{z}=0$.

As shown in Ref. [68], the measured velocity profiles across the channel with a parabolic fit are observed and recorded. Therefore, in this work, it is assumed that the nonlinear velocity profiles near the wall are parabolic in the $\bar{z}$ direction, conforming to the quadratic term fitting. The velocity profiles can be linear when the quadratic coefficient is 0. Then, the function of velocity profiles near the wall will be simplified as follows:

(42) $$u_{x}(\bar{x},\bar{z})=u_{x}(\bar{x})+\alpha_{1}{\bar{z}}^{2}+\beta_{1}\bar{z}+e_{1}$$

(43) $$u_{y}(\bar{y},\bar{z})=u_{y}(\bar{y})+\alpha_{2}{\bar{z}}^{2}+\beta_{2}\bar{z}+e_{2}$$

The acceleration can be approximated by relations as follows [69]:

(44) $$a_{x}\approx -\nu \frac{\partial^{2}u_{x}(\bar{x},\bar{z})}{\partial {\bar{z}}^{2}},a_{y}\approx -\nu \frac{\partial^{2}u_{y}(\bar{y},\bar{z})}{\partial {\bar{z}}^{2}}.$$

Then, the coefficients can be obtained by the acceleration, *α*_1_ = −0.5 *a_x_*/*ν*, *α*_2_ = −0.5 *a_y_*/*ν.*

The slip velocity can be expressed as:

(45) $$u_{sx}=u_{x}(\bar{x},\bar{z}){|}_{\bar{z}=1}-U_{Wx},u_{sy}=u_{y}(\bar{y},\bar{z}){|}_{\bar{z}=1}-U_{Wy}.$$

The slip velocity on a wall is characterized in the form of a Navier slip boundary condition in both the streamwise direction and the spanwise direction [35]:

(46) $$u_{sx}=b_{x}\frac{\partial u_{x}}{\partial z}{|}_{\mathrm{wall}}\;,\;u_{sy}=b_{y}\frac{\partial u_{y}}{\partial z}{|}_{\mathrm{wall}}$$

Considering Equations (42) and (43), (46) can be written as:

(47) $$u_{sx}=b_{x}\frac{\partial u_{x}}{\partial \bar{z}}{|}_{\bar{z}=0}=b_{x}\beta_{1},u_{sy}=b_{y}\frac{\partial u_{y}}{\partial \bar{z}}{|}_{\bar{z}=0}=b_{y}\beta_{2}.$$

With the Taylor expansion around the $\bar{z}$ = 1 in the local coordinate system, $u_{x}(\bar{z})$ and $u_{y}(\bar{z})$ can be approximated,

(48) $$u_{x}(\bar{z}){|}_{\bar{z}=2}=u_{x}(\bar{z}){|}_{\bar{z}=1}+\Delta \bar{z}\frac{\partial u_{x}(\bar{z})}{\partial \bar{z}}{|}_{\bar{z}=1}+\frac{\Delta {\bar{z}}^{2}}{2}(\frac{\partial^{2}u_{x}(\bar{z})}{\partial {\bar{z}}^{2}}){|}_{\bar{z}=1}+O(\Delta {\bar{z}}^{3})$$

(49) $$u_{y}(\bar{z}){|}_{\bar{z}=2}=u_{y}(\bar{z}){|}_{\bar{z}=1}+\Delta \bar{z}\frac{\partial u_{y}(\bar{z})}{\partial \bar{z}}{|}_{\bar{z}=1}+\frac{\Delta {\bar{z}}^{2}}{2}(\frac{\partial^{2}u_{y}(\bar{z})}{\partial {\bar{z}}^{2}}){|}_{\bar{z}=1}+O(\Delta {\bar{z}}^{3})$$

With the assumption of parabolic velocity profiles near the wall, substitute the above equations, and the following equations can be obtained,

(50) $$\begin{array}{l} u_{x}(\bar{x},\bar{z}){|}_{\bar{z}=2}-u_{x}(\bar{x},\bar{z}){|}_{\bar{z}=1}=(\Delta {\bar{z}}^{2}+2\Delta \bar{z})\alpha_{1}+\Delta \bar{z}\beta_{1},\\ u_{y}(\bar{y},\bar{z}){|}_{\bar{z}=2}-u_{x}(\bar{y},\bar{z}){|}_{\bar{z}=1}=(\Delta {\bar{z}}^{2}+2\Delta \bar{z})\alpha_{2}+\Delta \bar{z}\beta_{2}. \end{array}$$

Then, the relations between the coefficients are given as,

(51) $$\beta_{1}=\frac{\left(3-\Delta {\bar{z}}^{2}-2\Delta \bar{z}\right)}{\Delta \bar{z}-1+O(\Delta \bar{z})}\alpha_{1}$$

(52) $$\beta_{2}=\frac{\left(3-\Delta {\bar{z}}^{2}-2\Delta \bar{z}\right)}{\Delta \bar{z}-1+O(\Delta \bar{z})}\alpha_{2}$$

For D3Q19 lattice model, with $\rho u=\sum_{\alpha =0}^{18}\xi_{\alpha }f_{\alpha }$ known, the local velocities $u_{x},u_{y}$ at $\bar{z}$ = 1 and 2 can be calculated as,

(53) $$\begin{array}{l} \rho u_{x}{|}_{\bar{z}=1}={\tilde{f}}_{1}^{1}-{\tilde{f}}_{2}^{1}+{\tilde{f}}_{7}^{1}-{\tilde{f}}_{10}^{1}+{\tilde{f}}_{9}^{1}-{\tilde{f}}_{8}^{1},\\ \rho u_{x}{|}_{\bar{z}=2}={\tilde{f}}_{1}^{2}-{\tilde{f}}_{2}^{2}+{\tilde{f}}_{7}^{2}-{\tilde{f}}_{10}^{2}+{\tilde{f}}_{9}^{2}-{\tilde{f}}_{8}^{2}, \end{array}$$

(54) $$\begin{array}{l} \rho u_{y}{|}_{\bar{z}=1}={\tilde{f}}_{6}^{1}-{\tilde{f}}_{5}^{1}+{\tilde{f}}_{13}^{1}-{\tilde{f}}_{11}^{1}+{\tilde{f}}_{12}^{1}-{\tilde{f}}_{14}^{1},\\ \rho u_{y}{|}_{\bar{z}=2}={\tilde{f}}_{6}^{2}-{\tilde{f}}_{5}^{2}+{\tilde{f}}_{13}^{2}-{\tilde{f}}_{11}^{2}+{\tilde{f}}_{12}^{2}-{\tilde{f}}_{14}^{2}. \end{array}$$

Combined with the proposed slip boundary condition, the following relations can be obtained:

(55) $$\begin{array}{l} {\tilde{f}}_{9}-{\tilde{f}}_{8}={\tilde{f}}_{7}-{\tilde{f}}_{10}+{\sigma^{\prime }}_{x}\left\{\rho U_{Wx}-[{\tilde{f}}_{1}-{\tilde{f}}_{2}+2({\tilde{f}}_{7}-{\tilde{f}}_{10})]\right\},\\ {\tilde{f}}_{12}-{\tilde{f}}_{14}={\tilde{f}}_{13}-{\tilde{f}}_{11}+{\sigma^{\prime }}_{y}\left\{\rho U_{Wy}-[{\tilde{f}}_{6}-{\tilde{f}}_{5}+2({\tilde{f}}_{13}-{\tilde{f}}_{11})]\right\}. \end{array}$$

Then,

(56) $$\begin{array}{l} \rho u_{x}{|}_{\bar{z}=1}=(1-{\sigma^{\prime }}_{x})[{\tilde{f}}_{{}_{1}}^{1}-{\tilde{f}}_{{}_{2}}^{1}+2({\tilde{f}}_{{}_{7}}^{1}-{\tilde{f}}_{{}_{10}}^{1})]+{\sigma^{\prime }}_{x}\rho U_{Wx},\\ \rho u_{x}{|}_{\bar{z}=2}=(1-{\sigma^{\prime }}_{x})[{\tilde{f}}_{{}_{1}}^{2}-{\tilde{f}}_{{}_{2}}^{2}+2({\tilde{f}}_{{}_{7}}^{2}-{\tilde{f}}_{{}_{10}}^{2})]+{\sigma^{\prime }}_{x}\rho U_{Wx},\\ \rho u_{y}{|}_{\bar{z}=1}=(1-{\sigma^{\prime }}_{y})[{\tilde{f}}_{{}_{6}}^{1}-{\tilde{f}}_{{}_{5}}^{1}+2({\tilde{f}}_{{}_{13}}^{1}-{\tilde{f}}_{{}_{11}}^{1})]+{\sigma^{\prime }}_{y}\rho U_{Wy},\\ \rho u_{y}{|}_{\bar{z}=2}=(1-{\sigma^{\prime }}_{y})[{\tilde{f}}_{{}_{6}}^{2}-{\tilde{f}}_{{}_{5}}^{2}+2({\tilde{f}}_{{}_{13}}^{2}-{\tilde{f}}_{{}_{11}}^{2})]+{\sigma^{\prime }}_{y}\rho U_{Wy}. \end{array}$$

The difference value between velocity at $\bar{z}$ = 1 and $\bar{z}$ = 2 can be written as:

(57) $$\begin{array}{l} u_{x}{|}_{\bar{z}=2}-u_{x}{|}_{\bar{z}=1}=\frac{1}{\rho }(1-{\sigma^{\prime }}_{x})\{({\tilde{f}}_{{}_{1}}^{2}-{\tilde{f}}_{{}_{2}}^{2})-({\tilde{f}}_{{}_{1}}^{1}-{\tilde{f}}_{{}_{2}}^{1})+2[({\tilde{f}}_{{}_{7}}^{2}-{\tilde{f}}_{{}_{10}}^{2})-({\tilde{f}}_{{}_{7}}^{1}-{\tilde{f}}_{{}_{10}}^{1})]\},\\ u_{y}{|}_{\bar{z}=2}-u_{y}{|}_{\bar{z}=1}=\frac{1}{\rho }(1-{\sigma^{\prime }}_{y})\{({\tilde{f}}_{{}_{6}}^{2}-{\tilde{f}}_{{}_{5}}^{2})-({\tilde{f}}_{{}_{6}}^{1}-{\tilde{f}}_{{}_{5}}^{1})+2[({\tilde{f}}_{{}_{13}}^{2}-{\tilde{f}}_{{}_{11}}^{2})+({\tilde{f}}_{{}_{13}}^{1}-{\tilde{f}}_{{}_{11}}^{1})]\}. \end{array}$$

Inspired by Guo et al. [69], the collision and streaming rule in the LBM is adopted to establish the relationship between velocities near the wall. Considering the collision and streaming rule of LBE with BGK operator [70], the following relations can be obtained:

(58) $$x\;\text{-}\;\mathrm{direction}:{\tilde{f}}_{7}^{1}-{\tilde{f}}_{10}^{1}={\tilde{\bar{f}}}_{7}^{2}-{\tilde{\bar{f}}}_{10}^{2},{\tilde{f}}_{9}^{2}-{\tilde{f}}_{8}^{2}={\tilde{\bar{f}}}_{9}^{1}-{\tilde{\bar{f}}}_{8}^{1}$$

(59) $$y\;\text{-}\;\mathrm{direction}:{\tilde{f}}_{13}^{1}-{\tilde{f}}_{11}^{1}={\tilde{\bar{f}}}_{13}^{2}-{\tilde{\bar{f}}}_{11}^{2},{\tilde{f}}_{12}^{2}-{\tilde{f}}_{14}^{2}={\tilde{\bar{f}}}_{12}^{1}-{\tilde{\bar{f}}}_{14}^{1}$$

where $\tilde{\bar{f}}$ denotes the tracked distribution function in the collision.

Then, Equation (57) can be simplified as follows:

(60) $$u_{x}{|}_{\bar{z}=2}-u_{x}{|}_{\bar{z}=1}=\frac{3{\sigma^{\prime }}_{x}}{\tau (1-{\sigma^{\prime }}_{x})}(u_{x}{|}_{\bar{z}=1}-U_{Wx})$$

(61) $$u_{y}{|}_{\bar{z}=2}-u_{y}{|}_{\bar{z}=1}=\frac{3{\sigma^{\prime }}_{y}}{\tau (1-{\sigma^{\prime }}_{y})}(u_{y}{|}_{\bar{z}=1}-U_{Wy})$$

The slip velocity could be calculated as:

(62) $$u_{sx}=b_{x}\frac{\partial u_{x}}{\partial \bar{z}}{|}_{\bar{z}=0}=b_{x}\beta_{1}=u_{x}{|}_{\bar{z}=1}-U_{Wx}=\frac{\tau (1-{\sigma^{\prime }}_{x})}{3{\sigma^{\prime }}_{x}}(u_{x}{|}_{\bar{z}=2}-u_{x}{|}_{\bar{z}=1})$$

(63) $$u_{sy}=b_{x}\frac{\partial u_{y}}{\partial \bar{z}}{|}_{\bar{z}=0}=b_{y}\beta_{1}=u_{y}{|}_{\bar{z}=1}-U_{Wy}=\frac{\tau (1-{\sigma^{\prime }}_{y})}{3{\sigma^{\prime }}_{y}}(u_{y}{|}_{\bar{z}=2}-u_{y}{|}_{\bar{z}=1})$$

Finally, the relations between the slip lengths and parameters are obtained:

(64) $$b_{x}=\frac{\tau (1-{\sigma^{\prime }}_{x})}{3{\sigma^{\prime }}_{x}}(u_{x}{|}_{\bar{z}=2}-u_{x}{|}_{\bar{z}=1})/\beta_{1}=(\Delta {\bar{z}}^{2}+2\Delta \bar{z})\frac{\alpha_{1}}{\beta_{1}}+\Delta \bar{z}$$

(65) $$b_{y}=\frac{\tau (1-{\sigma^{\prime }}_{y})}{3{\sigma^{\prime }}_{y}}(u_{y}{|}_{\bar{z}=2}-u_{y}{|}_{\bar{z}=1})/\beta_{1}=(\Delta {\bar{z}}^{2}+2\Delta \bar{z})\frac{\alpha_{2}}{\beta_{2}}+\Delta \bar{z}$$

With the known values of $\alpha_{1}/\beta_{1},\alpha_{2}/\beta_{2}$, Equations (64) and (65) can be simplified,

(66) $${\sigma^{\prime }}_{x}=\frac{1}{1+\frac{3b_{x}}{A\tau }},{\sigma^{\prime }}_{y}=\frac{1}{1+\frac{3b_{y}}{A\tau }}.$$

where A denotes the correction coefficient and is determined by:

(67) $$A=(\Delta {\bar{z}}^{2}+\Delta \bar{z})\frac{\Delta \bar{z}-1+O(\Delta {\bar{z}}^{3})}{3-\Delta {\bar{z}}^{2}-2\Delta \bar{z}}+\Delta \bar{z}$$

where $\Delta \bar{z}$ is the lattice grid spacing.

For the upper horizontal wall boundary in 3D, a new slip boundary condition is significantly determined by Equations (35), (66), and (67). Similar derivation and operation can be applied to the lower wall and side walls.

### 2.5. Corner Boundary Condition

The above discussion on boundary conditions focuses on straight surfaces. Considering the singularity, the treatment of corners should not be ignored in numerical simulations of the flow, such as the lid-driven cavity flow. Although corners account for only a few nodes, these corners should not be underestimated because the particle can stream in the fluid domain, which has influences on the performance of the numerical simulation. One single point may contaminate the numerical solution everywhere [71]. One of the earliest systematic approaches to treating corners in DUGKS was proposed by Guo et al. [72]. However, this approach is limited to 2D implementations. In this work, to reduce the singularities and improve the performance of numerical simulation, an approach to treating the corner boundary condition is proposed for the DUGKS based on the D3Q19 model with 19 independent moments [73].

(68) $$\begin{array}{l} 0^{\mathrm{th}}:\rho =\sum_{i}f_{i};\;1^{\mathrm{st}}:\rho u_{\alpha }=\sum_{i}f_{i}\xi_{i\alpha };\;2^{\mathrm{nd}}:\Pi_{\alpha \beta }=\sum_{i}f_{i}\xi_{i\alpha }\xi_{i\beta };\\ 3^{\mathrm{rd}}:Q_{\alpha \beta \gamma }=\sum_{i}f_{i}\xi_{i\alpha }\xi_{i\beta }\xi_{i\gamma };\;{\;4}^{\mathrm{th}}:S_{\alpha \beta \gamma \delta }=\sum_{i}f_{i}\xi_{i\alpha }\xi_{i\beta }\xi_{i\gamma }\xi_{i\delta }. \end{array}$$

The 0th moment has 1 equation, the 1st moment has 3 equations, the 2nd moment has 6 equations, the 3rd moment has 6 equations, and the 4th moment has 3 equations. In the 3D domain, there are 12 unknowns at every corner. So, 12 linearly independent moments are required. For the D3Q19 model, as shown in Figure 1b, the unknown functions are $f_{1},f_{3},f_{6},f_{7},f_{9},f_{10},f_{11},f_{12},f_{13},f_{15},f_{16},f_{17}$, considering the low-south-west corner. We select the momenta $\rho u_{x},\rho u_{y},\rho u_{z}$, the momentum fluxes and shear stresses $\Pi_{xx},\Pi_{yy},\Pi_{zz},\Pi_{xy},\Pi_{xz},\Pi_{yz}$, and three higher-order moments $Q_{xxy},Q_{yyz},Q_{xzz}$ as 12 linearly independent moments.

(69) $$\mathrm{rank}\left[\begin{array}{cc} {\tilde{f}}_{1}+{\tilde{f}}_{9}-{\tilde{f}}_{10}+{\tilde{f}}_{15}-{\tilde{f}}_{16}+{\tilde{f}}_{17} & \rho u_{x}\\ {\tilde{f}}_{6}-{\tilde{f}}_{11}+{\tilde{f}}_{12}+{\tilde{f}}_{13}-{\tilde{f}}_{15}+{\tilde{f}}_{16}+{\tilde{f}}_{17} & \rho u_{y}\\ {\tilde{f}}_{3}+{\tilde{f}}_{7}-{\tilde{f}}_{9}+{\tilde{f}}_{10}+{\tilde{f}}_{11}-{\tilde{f}}_{12}+{\tilde{f}}_{13} & \rho u_{z}\\ {\tilde{f}}_{1}+{\tilde{f}}_{9}+{\tilde{f}}_{10}+{\tilde{f}}_{15}+{\tilde{f}}_{16}+{\tilde{f}}_{17} & \Pi_{xx}\\ {\tilde{f}}_{6}+{\tilde{f}}_{11}+{\tilde{f}}_{12}+{\tilde{f}}_{13}+{\tilde{f}}_{15}+{\tilde{f}}_{16}+{\tilde{f}}_{17} & \Pi_{yy}\\ {\tilde{f}}_{3}+{\tilde{f}}_{7}+{\tilde{f}}_{9}+{\tilde{f}}_{10}+{\tilde{f}}_{11}+{\tilde{f}}_{12}+{\tilde{f}}_{13} & \Pi_{zz}\\ {\tilde{f}}_{17}-{\tilde{f}}_{15}-{\tilde{f}}_{16} & \Pi_{xy}\\ {\tilde{f}}_{7}-{\tilde{f}}_{9}-{\tilde{f}}_{10} & \Pi_{xz}\\ {\tilde{f}}_{13}-{\tilde{f}}_{11}-{\tilde{f}}_{12} & \Pi_{yz}\\ {\tilde{f}}_{17}-{\tilde{f}}_{15}+{\tilde{f}}_{16} & Q_{xxy}\\ {\tilde{f}}_{7}+{\tilde{f}}_{9}-{\tilde{f}}_{10} & Q_{xzz}\\ {\tilde{f}}_{13}+{\tilde{f}}_{11}-{\tilde{f}}_{12} & Q_{yyz} \end{array}\right]=12$$

The moments can be approximated by the Chapman–Enskog expansion as follows:

(70) $$\Pi_{\alpha \beta }=\Pi_{\alpha \beta }^{\left(0\right)}+\varepsilon \Pi_{\alpha \beta }^{\left(1\right)}+O(\varepsilon^{2})\approx \Pi_{\alpha \beta }^{\left(0\right)},Q_{\alpha \beta }=Q_{\alpha \beta }^{\left(0\right)}+\varepsilon Q_{\alpha \beta }^{\left(1\right)}+O(\varepsilon^{2})\approx Q_{\alpha \beta }^{\left(0\right)}$$

where the equilibrium part of the momentum flux tensor ($\Pi_{\alpha \beta }^{\left(0\right)}$) and the third-order moment ($Q_{\alpha \beta }^{\left(0\right)}$) can be expressed as:

(71) $$\begin{array}{l} \Pi_{\alpha \beta }^{\left(0\right)}=\sum_{i}f_{i}^{\left(0\right)}\xi_{i\alpha }\xi_{i\beta }=\rho u_{\alpha }u_{\beta }+\rho c_{s}^{2}\delta_{\alpha \beta },\\ Q_{\alpha \beta \gamma }^{\left(0\right)}=\sum_{i}f_{i}^{\left(0\right)}\xi_{i\alpha }\xi_{i\beta }\xi_{i\gamma }=\rho c_{s}^{2}(u_{\alpha }\delta_{\beta \gamma }+u_{\beta }\delta_{\alpha \gamma }+u_{\gamma }\delta_{\alpha \beta }). \end{array}$$

The velocity is set to 0 at the corner, and some terms are assumed to be negligible. The momentum fluxes and shear stresses $\Pi_{xx},\Pi_{yy},\Pi_{zz},\Pi_{xy},\Pi_{xz},\Pi_{yz}$, and three higher-order moments $Q_{xxy},Q_{yyz},Q_{xzz}$ are written as follows:

(72) $$\begin{array}{l} \Pi_{xx}=\Pi_{yy}=\Pi_{zz}=\rho c_{s}^{2}=\rho /3,\\ \Pi_{xy}=\rho u_{x}u_{y}=0,\Pi_{xz}=\rho u_{x}u_{z}=0,\Pi_{yz}=\rho u_{y}u_{z}=0,\\ Q_{xxy}=\frac{\rho }{3}(u_{x}\delta_{xy}+u_{x}\delta_{xy}+u_{y}\delta_{xx})=\frac{\rho }{3}u_{y}=0,\\ Q_{yyz}=\frac{\rho }{3}(u_{y}\delta_{yz}+u_{y}\delta_{yz}+u_{z}\delta_{yy})=\frac{\rho }{3}u_{z}=0,\\ Q_{xzz}=\frac{\rho }{3}(u_{x}\delta_{zz}+u_{x}\delta_{xz}+u_{z}\delta_{xz})=\frac{\rho }{3}u_{x}=0. \end{array}$$

The unknown functions are calculated as:

(73) $$\begin{array}{l} {\tilde{f}}_{1}=-\frac{\rho }{3}+{\tilde{f}}_{2}+2{\tilde{f}}_{5}+4{\tilde{f}}_{14}+4{\tilde{f}}_{18}\\ {\tilde{f}}_{3}=-\frac{\rho }{3}+{\tilde{f}}_{4}+2{\tilde{f}}_{2}+4{\tilde{f}}_{8}+4{\tilde{f}}_{18}\\ {\tilde{f}}_{6}=-\frac{\rho }{3}+{\tilde{f}}_{5}+2{\tilde{f}}_{4}+4{\tilde{f}}_{8}+4{\tilde{f}}_{14}\\ {\tilde{f}}_{7}=\frac{\rho }{6}-{\tilde{f}}_{2}-{\tilde{f}}_{8}-2{\tilde{f}}_{18}\\ {\tilde{f}}_{9}={\tilde{f}}_{8}\\ {\tilde{f}}_{10}=\frac{\rho }{6}-{\tilde{f}}_{2}-{\tilde{f}}_{8}-2{\tilde{f}}_{18}\\ {\tilde{f}}_{11}={\tilde{f}}_{14}\\ {\tilde{f}}_{12}=\frac{\rho }{6}-{\tilde{f}}_{4}-{\tilde{f}}_{14}-2{\tilde{f}}_{8}\\ {\tilde{f}}_{13}=\frac{\rho }{6}-{\tilde{f}}_{4}-{\tilde{f}}_{14}-2{\tilde{f}}_{8}\\ {\tilde{f}}_{15}=\frac{\rho }{6}-{\tilde{f}}_{5}-{\tilde{f}}_{18}-2{\tilde{f}}_{14}\\ {\tilde{f}}_{16}={\tilde{f}}_{18}\\ {\tilde{f}}_{17}=\frac{\rho }{6}-{\tilde{f}}_{5}-{\tilde{f}}_{18}-2{\tilde{f}}_{14} \end{array}$$

The density at the low-south-west corner is calculated as:

(74) $$\rho ={\tilde{f}}_{0}+2({\tilde{f}}_{2}+{\tilde{f}}_{4}+{\tilde{f}}_{5})+4({\tilde{f}}_{8}+{\tilde{f}}_{14}+{\tilde{f}}_{18}).$$

Similar manipulations can be applied to other corner nodes.

### 2.6. Algorithm

The updating of ${\tilde{f}}_{\alpha }$ from time $t^{n}=n\Delta t$ to time $t^{n+1}=\left(n+1\right)\Delta t$ in the DUGKS can be performed as the following brief algorithm.

1. Initialize the density, velocity, and viscosity. Obtain the values of $f_{\alpha }^{eq,n}$ and ${\tilde{f}}_{\alpha }^{n}$ at time *t* = 0.
2. Compute the distribution functions ${\bar{f}}_{\alpha }^{+,n}$ and ${\tilde{f}}_{\alpha }^{+,n}$ using Equations (16) and (18).
3. Compute the value of $\nabla {\bar{f}}_{\alpha }^{+,n}\left(x_{b}\right)$ and ${\bar{f}}_{\alpha }^{+,n}\left(x_{b}\right)$ using Equation (15).
4. Compute the distribution function ${\bar{f}}_{\alpha }^{n+1/2}\left(x_{b}\right)$ using Equations (14) and (13).
5. Get the macro values of density and velocity using Equation (17). Compute the equilibrium distribution function $f_{\alpha }^{eq,n+1/2}\left(x_{b}\right)\;$.
6. Compute the distribution function $f_{\alpha }^{n+1/2}\left(x_{b}\right)$ using Equation (13). Obtain the flux ${ℱ}_{\alpha }^{n+1/2}$ by Equation (5).
7. For the unknown distribution functions at the boundary or corner, the boundary conditions are employed, such as Equations (35) or (73).
8. Update the distribution function ${\tilde{f}}_{\alpha }^{n+1}$ using Equation (8). Obtain the macro values of density and velocity.
9. Repeat steps (2)–(8) until the convergence criterion is satisfied.

In C++ DUGKS computer code, the convergence criterion for attaining the steady-state solution is $\sum \left|u\left(t\right)-u\left(t-1000\Delta t\right)\right|/\sum \left|u\left(t\right)\right|<10^{-6}$, where u(t) represents the velocity in the flow field.

## 3. Numerical Validation

The flow in a 3D channel is a fundamental case in science and engineering. Only a few references on the anisotropic slip boundary condition are available for comparison, so the flow in the 3D channel is selected as the case for numerical validation.

### 3.1. Comparison with Single-Component Lattice Boltzmann Simulation

**Figure 3:**
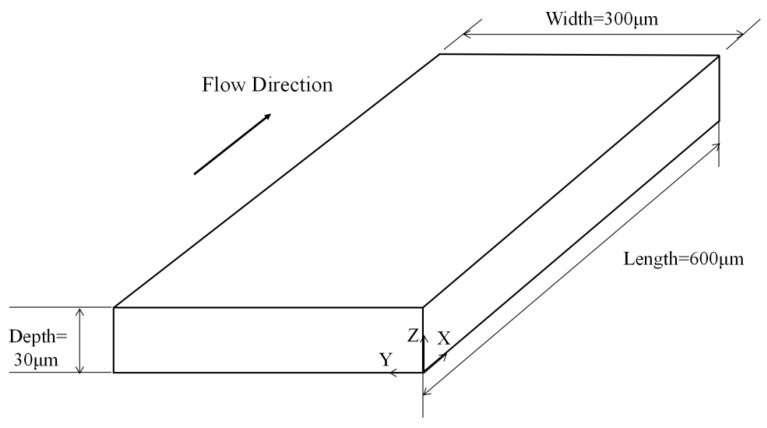
The diagram of a 3D microchannel.

In Ref. [11], the slip boundary condition is modelled by combining the bounce-back and specular reflection (BSR) scheme using the single-component lattice Boltzmann method. The relevant parameters in the present simulation remain the same as those in Ref. [11]. As shown in Figure 3, the microchannel’s length, width, and height are 600 μm, 300 μm, and 30 μm, respectively. With the grid convergence study, the spatial discretization with resolution 400 × 200 × 20 (X, Y, and Z directions, respectively) is selected for the subsequent numerical simulations. The inlet and outlet along the X-direction adopt the periodic boundary condition. The remaining four walls adopt the no-slip/slip boundary conditions. For the no-slip case, the bounce-back scheme in LBM is used without treating the corner in Ref. [11], and the bounce-back scheme in DUGKS is used with the corner boundary condition in the present work. For the slip case, the BSR scheme is employed in Ref. [11], and the present method is employed in this work.

**Figure 4:**
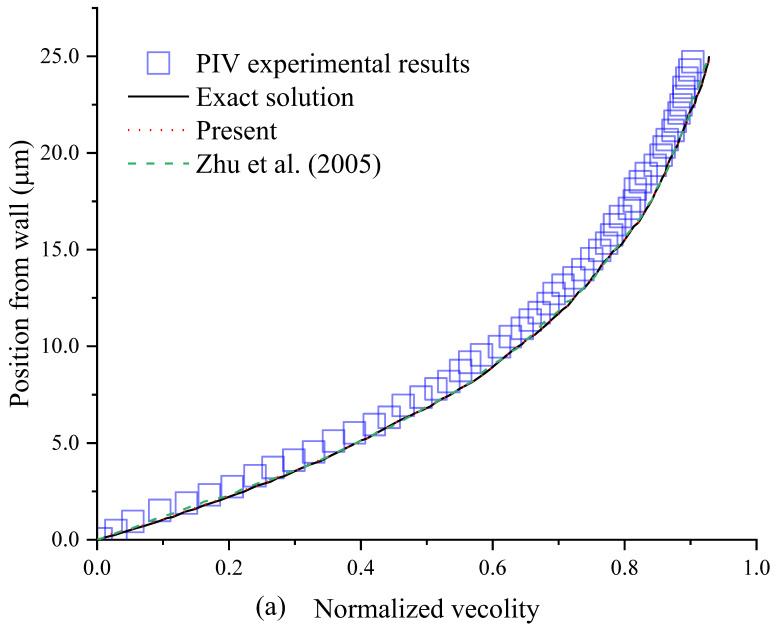

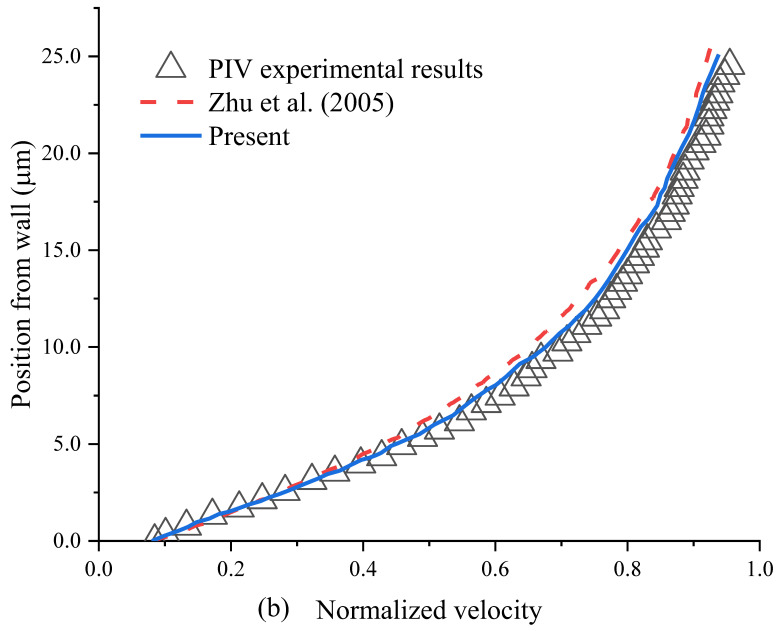
Velocity profiles in the no-slip case (**a**) and slip case (**b**). The data are obtained from the line (X = 300 μm, Z = 15 μm) normal to the right sidewall as a function of Y.

In the no-slip case, the present results agree well with the exact solution [74] and experimental result [9], as shown in Figure 4a. It is observed that the present results agree a little better with the exact solution than the BSR scheme in Ref. [11], which shows that the 3D corner boundary condition improves the accuracy.

In the slip case, the present results are closer to the experimental results [9] than those in Ref. [11], as shown in Figure 4b. The present method is more accurate than the BSR scheme in Ref. [11], which may be partly explained by the case that the BSR scheme can generate numerical slip, but the present method with the external force term can eliminate the numerical slip.

### 3.2. Comparison with Direct Numerical Simulation

In Ref. [14], the value of the streamwise slip length is set to equal the spanwise slip length. Furthermore, the case where the value of streamwise slip length is not equal to the spanwise slip length should be considered. With the different values of streamwise and spanwise slip lengths, the effect of anisotropic slip on velocity profiles and drag has been addressed using direct numerical simulations (DNS) of a turbulent channel flow [75]. In Ref. [75], the Navier slip length boundary condition adopts a linear slip length model. In this work, the present boundary condition is related to the second partial derivative of the velocity, with the assumption of nonlinear velocity profiles near the wall.

To test the accuracy of predicting the drag and velocity, the present method is performed in six different cases at Re_τ_ = *u*_τ0_*δ*/*ν* = 180: (1) Case 1: *b_x_*^+0^ = 0.1, *b_y_*^+0^ = 1; (2) Case 2: *b_x_*^+0^ = 0.316, *b_y_*^+0^ = 1; (3) Case 3: *b_x_*^+0^ = 3.16, *b_y_*^+0^ = 1; (4) Case 4: *b_x_*^+0^ = 10, *b_y_*^+0^ = 1; (5) Case 5: *b_x_*^+0^ = 0.631, *b_y_*^+0^ = 1; (6) Case 6: *b_x_*^+0^ = 2.51, *b_y_*^+0^ = 10. *δ* and *ν* denote the channel half-height and kinematic viscosity, respectively. *u*_τ0_ denotes the wall shear (friction) velocity in channel flow with no-slip walls. It is noted that the superscript +0 indicates slip length scales given in units of the viscous length scale *ν*/*u*_τ0_ in the respective no-slip reference case [76].

**Figure 5:**
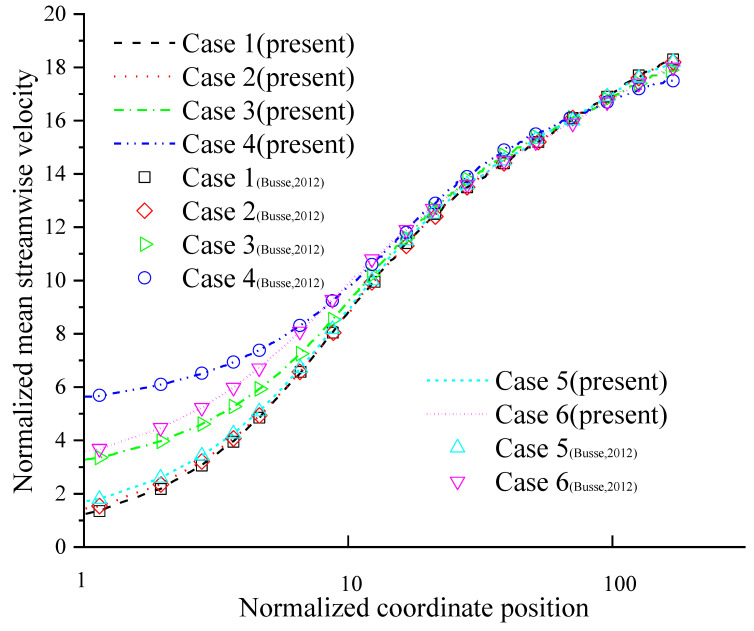
The mean streamwise velocity profiles.

**Figure 6:**
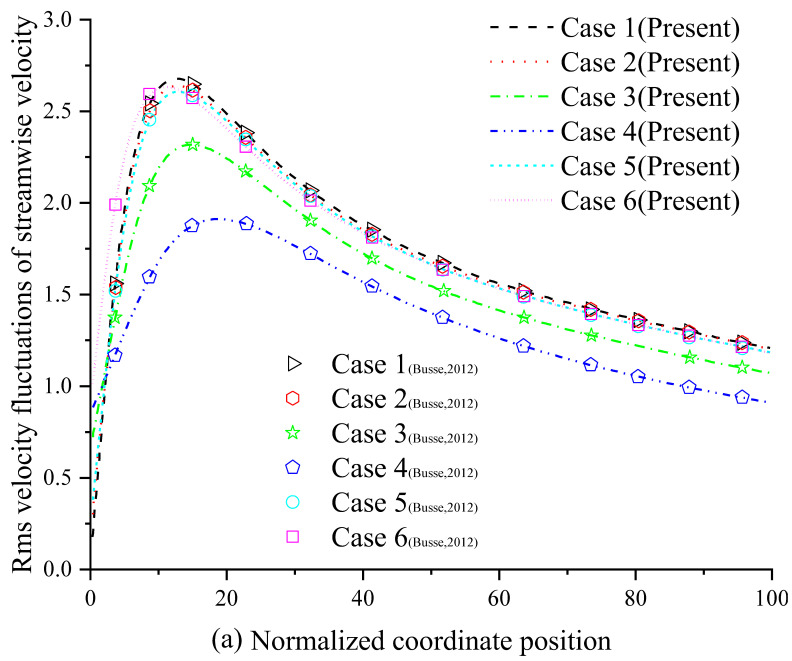

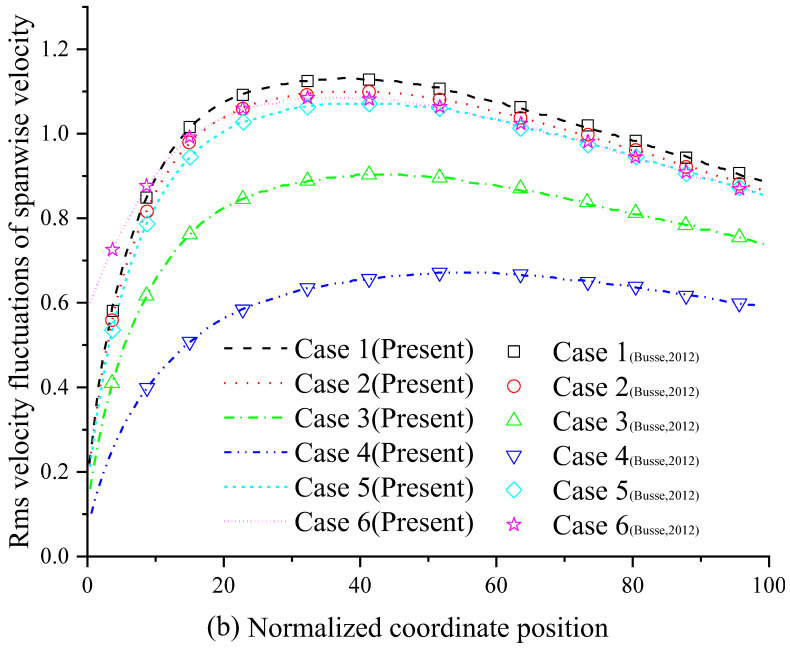
The rms velocity fluctuations of the streamwise velocity (**a**) and the spanwise velocity (**b**).

The numerical results of the present method are compared to the data in Ref. [75]. The mean streamwise velocity profile is shown in Figure 5, and the root-mean-square (rms) velocity fluctuations are shown in Figure 6. As shown in Figure 5 and Figure 6, the present method is also accurate in predicting the velocity profiles in a turbulent channel flow with an anisotropic slip wall. Similar conclusions to those reported by A. Busse and N. D. Sandham [75] are obtained: the streamwise slip length is mainly responsible for determining mean velocity profiles. Streamwise slip length always has a reducing effect on the intensity of the turbulent fluctuations, and the reducing effect will increase with increasing slip length. Finite streamwise slip length can limit the turbulence-intensifying effects of infinite spanwise slip, thereby limiting the adverse effects of spanwise slip.

To investigate the influence of an anisotropic slip on drag, the DUGKS simulations are conducted by adjusting the streamwise and spanwise slip lengths with the present slip boundary condition. The investigated slip length values are selected randomly. The present results are compared with those in Ref. [75].

The percentage change in drag (DD) is defined by DD = (*dp*/*dx*–*dp*/*dx*|_0_)/(*dp*/*dx*|_0_), where *dp*/*dx* and *dp*/*dx*|_0_ represent the mean streamwise pressure gradient in the present and reference case, respectively. If DD < 0, the drag is reduced. The DD values are obtained from numerical results in the case of Re_τ0_ = 180 based on friction velocity *u*_τ0_ in the reference case.

**Figure 7:**
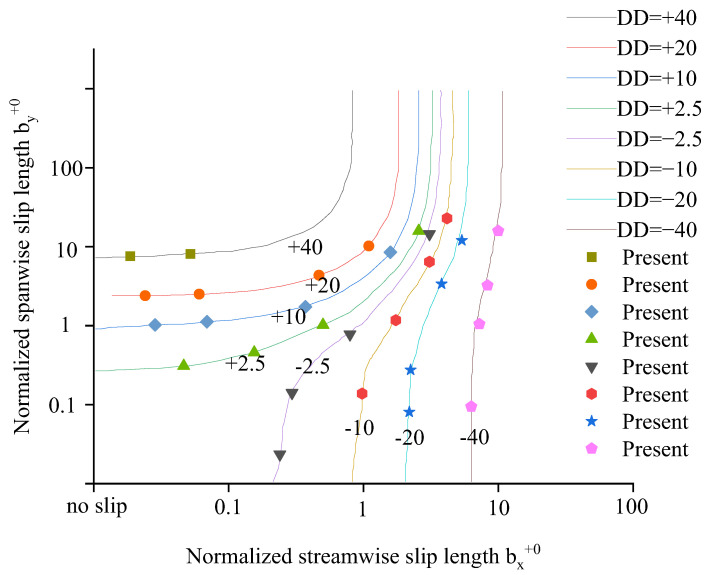
The percentage change in drag versus the streamwise and spanwise slip lengths. (Dots: present, Lines: DNS data [75]).

As shown in Figure 7, the dots match well with the lines, indicating that the present method is also accurate in predicting the change in drag. The same trends reported by Min and Kim [14] are recovered: drag is reduced in cases with pure streamwise slip and isotropic slip, but drag is increased in cases with pure spanwise slip.

## 4. Application to the Two-Sided Orthogonal Oscillating Micro-Lid-Driven Cavity Flow

### 4.1. Problem Description

**Figure 8:**
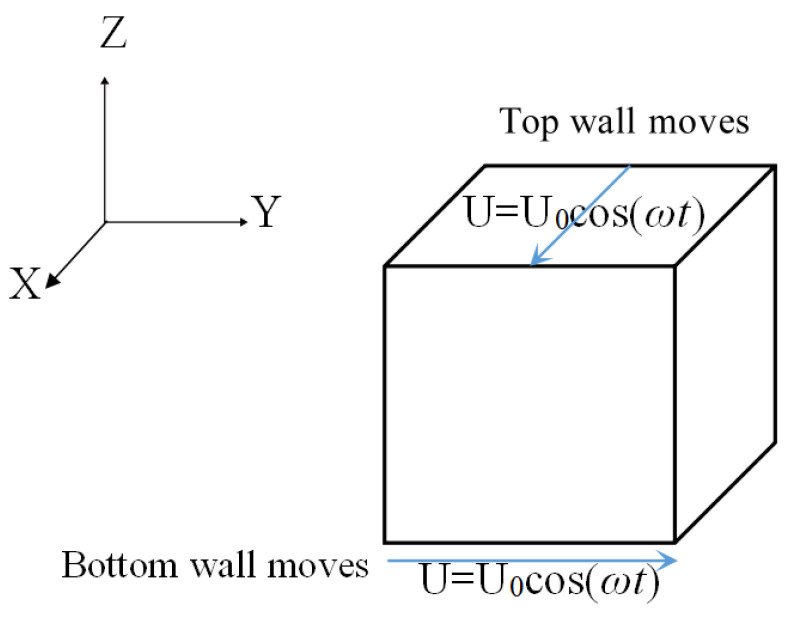
The two-sided orthogonal oscillating wall motion of the micro-lid-driven cavity.

The problem is the micro-lid-driven cavity flow with two moving walls, as shown in Figure 8. For two-sided oscillating wall motion, the top and bottom walls move with oscillating velocity U = U_0_cos(*ωt*), where U_0_ = 1.0 m/s, oscillating frequency *ω* = 1875 π/s. The directions of the top and bottom walls are positive X-direction and Y-direction, respectively. The size of the cavity is 400 μm × 400 μm × 400 μm. The cavity is filled with water. The density and kinematic viscosity of water are $\rho =1000\;\mathrm{kg}/m^{3}$ and $\upsilon =1.0\times 10^{-6}m^{2}/s$, respectively. The Reynolds number is calculated as Re = U_0_ × L/*ν* = 1.0 × 0.0004/10^−6^ = 400. For simplicity, *ω* has been dimensionalized as *ω’* = *ω*L/U_0_ = 0.75π, and St = *ω*L^2^/*ν* = *ω’*Re = 300π.

### 4.2. Convergence Validation Study

**Figure 9:**
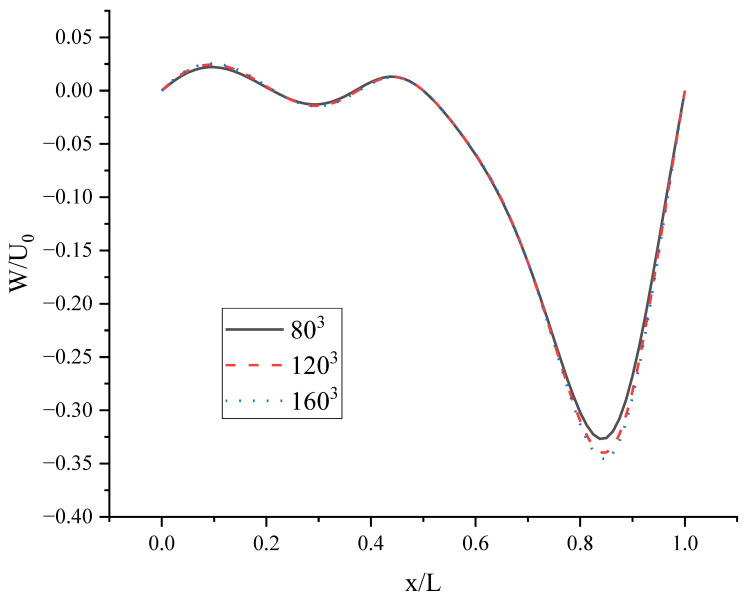
Velocity profiles for W on the horizontal centerlines of plane at y/L = 0.5.

To choose an optimal lattice size utilizing fewer computational resources, lattice size convergence is studied. Numerical simulations with two-sided uniformly moving wall motions are performed at Re = 400 using three lattice sizes: 80^3^ (coarse), 120^3^ (medium), and 160^3^ (fine). Figure 9 shows the negligible improvement in the velocity profile on increasing the lattice size from 120^3^ to 160^3^.

So, the spatial discretization with resolution 120 × 120 × 120 is used for performing subsequent numerical simulations with two-sided orthogonal oscillating wall motions. To keep Re = 400, U_0lattice_ = 1.0/15 and the kinematic viscosity is set to *ν*_lattice_ = 0.02. The present slip boundary condition is applied to the top and bottom wall, and the corner boundary condition is applied to four corner nodes in the cavity. The four side walls remain at rest with the no-slip boundary condition.

### 4.3. Results and Discussion

There are mainly two types of slip distribution: pure streamwise slip emerges on both the top and bottom wall surfaces; and both streamwise and spanwise slips emerge on the top wall surface. For comparison, fourteen cases are considered: (a) both top and bottom walls: *b_x_* = *b_y_* = 0; (b) top wall: *b_x_* = 0.1, *b_y_* = 0, bottom wall: *b_x_* = *b_y_* = 0; (c) top wall: *b_x_* = *b_y_* = 0, bottom wall: *b_x_* = 0,*b_y_* = 0.1; (d) top wall: *b_x_* = 0,*b_y_* = 0.1, bottom wall: *b_x_* = *b_y_* = 0; (e) top wall: *b_x_* = 0.1, *b_y_* = 0, bottom wall: *b_x_* = 0, *b_y_* = 0.1; (f) top wall: *b_x_* = 0.1,*b_y_* = 0.1, bottom wall: *b_x_* = *b_y_* = 0; (g) top wall: *b_x_* = 0.1, *b_y_* = 0, bottom wall: *b_x_* = 0,*b_y_* = 0.05; (h) top wall: *b_x_* = 0.1, *b_y_* = 0.05, bottom wall: *b_x_* = *b_y_* = 0; (i) top wall: *b_x_* = 0.05, *b_y_* = 0, bottom wall: *b_x_* = 0, *b_y_* = 0.1; (j) top wall: *b_x_* = 0.05, *b_y_* = 0.1, bottom wall: *b_x_* = *b_y_* = 0; (k) top wall: *b_x_* = 0.1, *b_y_* = 0, bottom wall: *b_x_* = 0, *b_y_* = 0.2; (l) top wall: *b_x_* = 0.1, *b_y_* = 0.2, bottom wall: *b_x_* = *b_y_* = 0; (m) top wall: *b_x_* = 0.2, *b_y_* = 0, bottom wall: *b_x_* = 0, *b_y_* = 0.1; (n) top wall: *b_x_* = 0.2, *b_y_* = 0.1, bottom wall: *b_x_* = *b_y_* = 0. In Ref. [9], their work yields a slip length of approximately 1 μm at the wall coated with hydrophobic octadecyltrichlorosilane for water flow. In the present work, considering actual value of slip length, the values of 0.05, 0.1, and 0.2 in the lattice unit correspond to 0.25 μm, 0.5 μm, and 1 μm, respectively. The symbols *b_x_* and *b_y_* represent the slip length in the X and Y directions, respectively.

The velocity components and vorticity are Important and common parameters to describe the flow. The present work performs a comprehensive parametric study discussing flow velocity components and vorticity. It is noted that U, V, and W are used to denote the velocity component in the X, Y, and Z directions, respectively. The vorticity magnitude is calculated as √{(∂W/∂Y-∂V/∂Z)^2^ + (∂U/∂Z-∂W/∂X)^2^ + (∂V/∂X-∂U/∂Y)^2^}.

**Figure 10:**
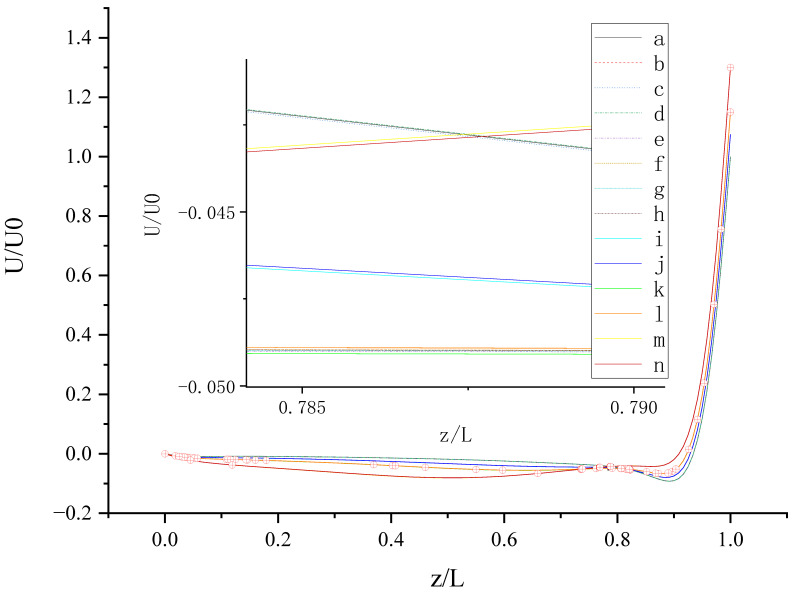
U along the centerline *Z*-axis at t = T. The red circles represent the points of intersection.

**Figure 11:**
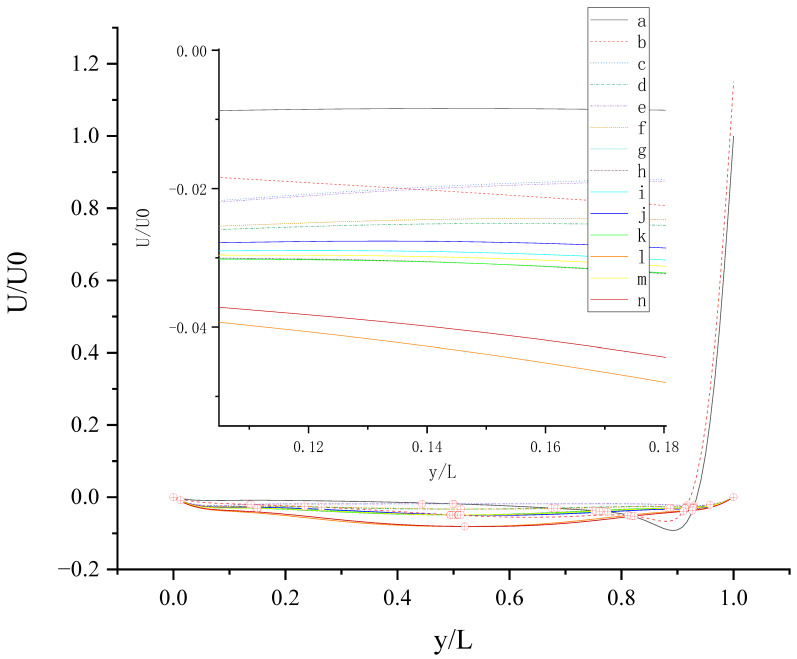
U along the centerline *Y*-axis at t = T. The red circles represent the points of intersection.

**Figure 12:**
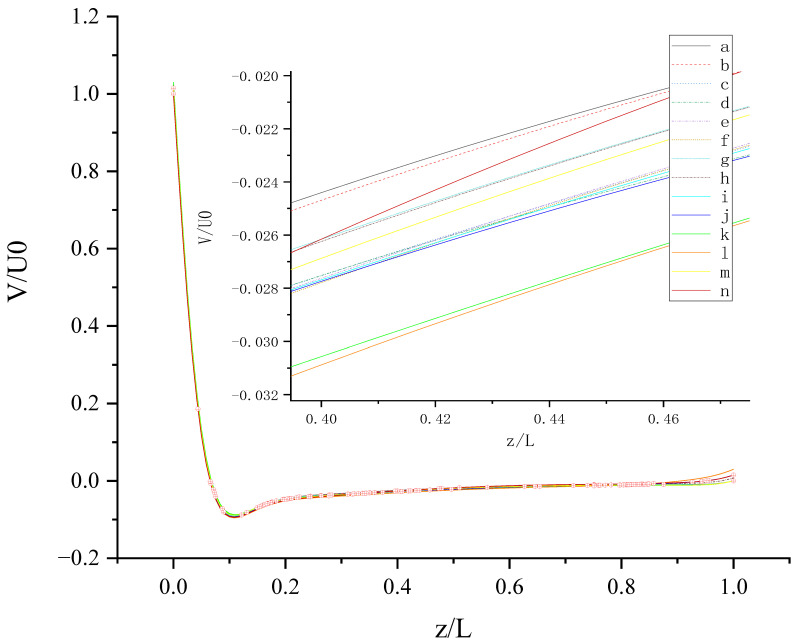
V along the centerline *Z*-axis at t = T. The red circles represent the points of intersection.

**Figure 13:**
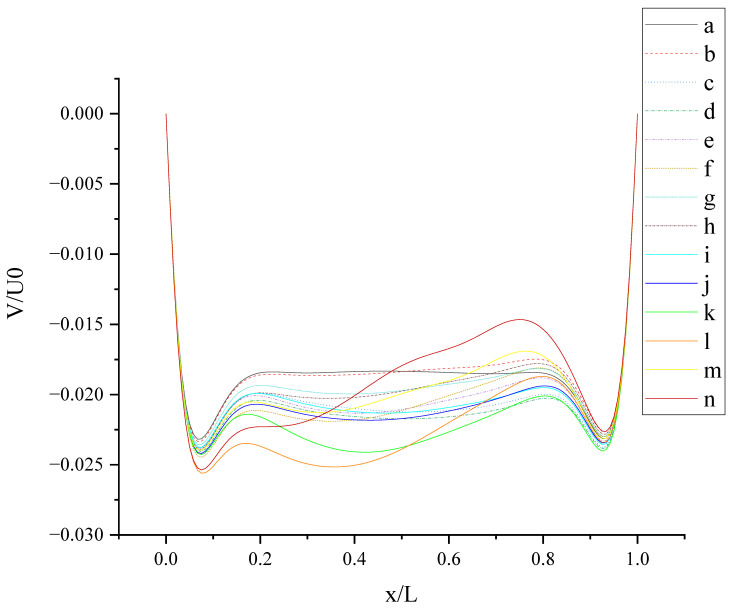
V along the centerline *X*-axis at t = T.

**Figure 14:**
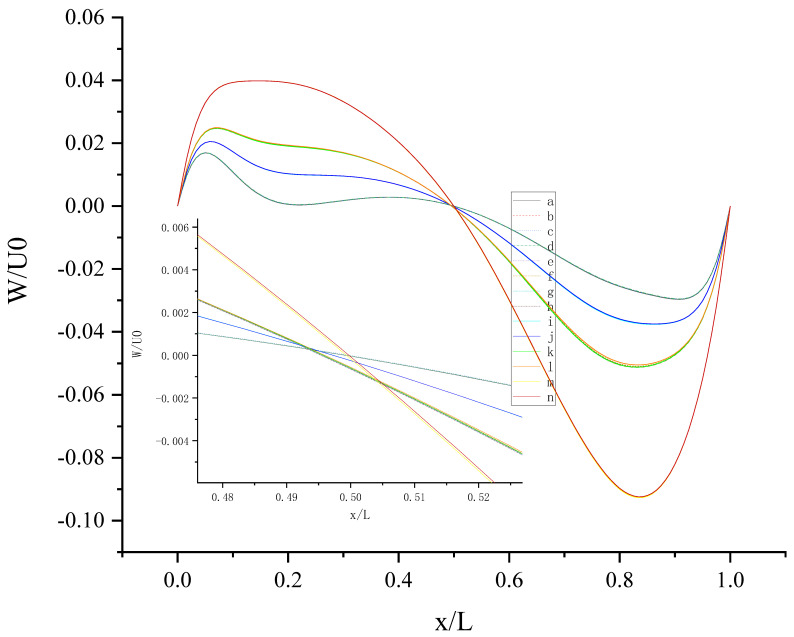
W along the centerline *X*-axis at t = T.

**Figure 15:**
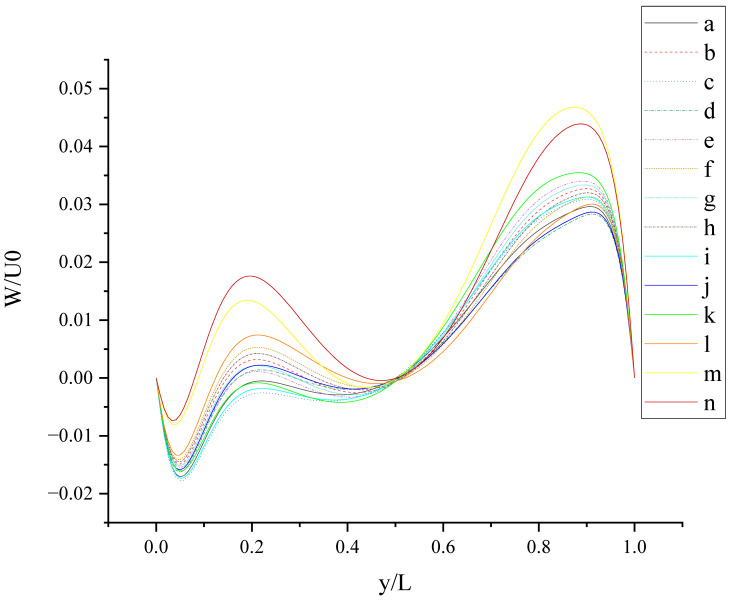
W along the centerline *Y*-axis at t = T.

**Figure 16:**
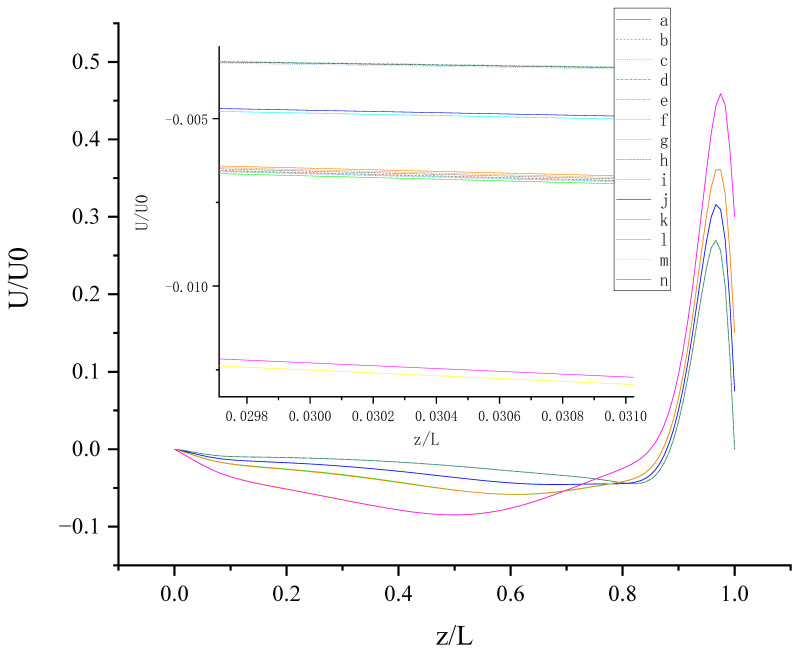
U along the centerline *Z*-axis at t = 0.25 T.

**Figure 17:**
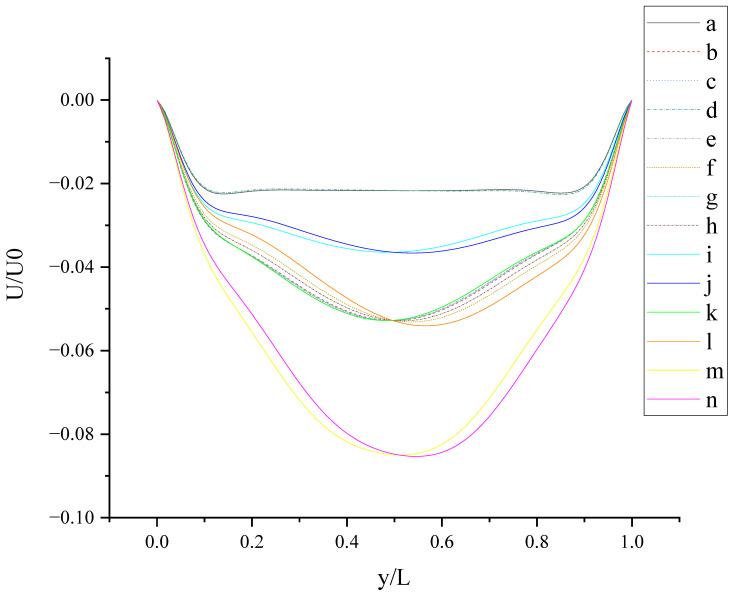
U along the centerline *Y*-axis at t = 0.25 T.

**Figure 18:**
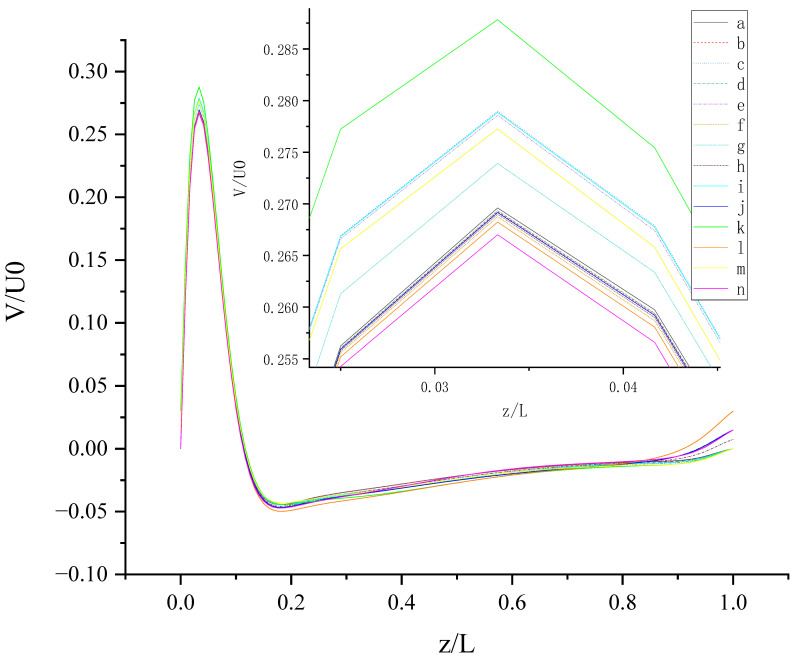
V along the centerline *Z*-axis at t = 0.25 T.

**Figure 19:**
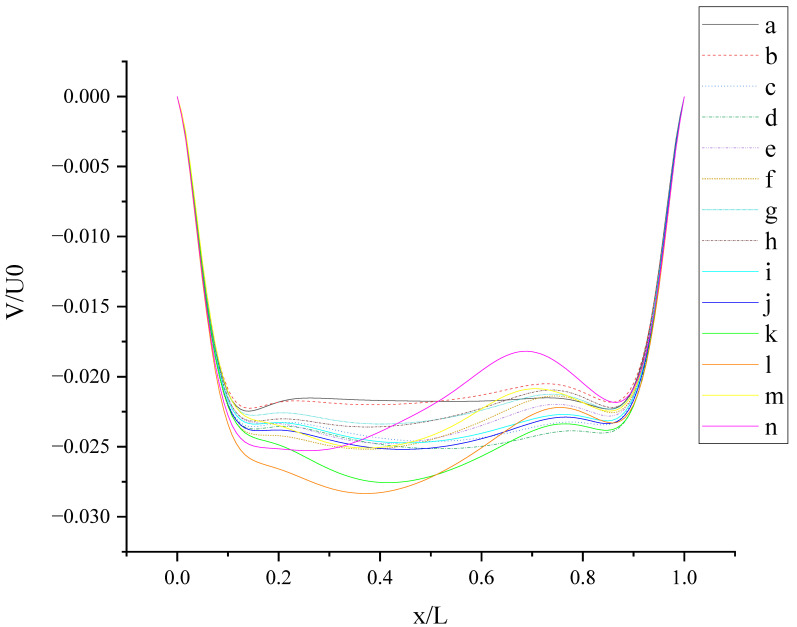
V along the centerline *X*-axis at t = 0.25 T.

**Figure 20:**
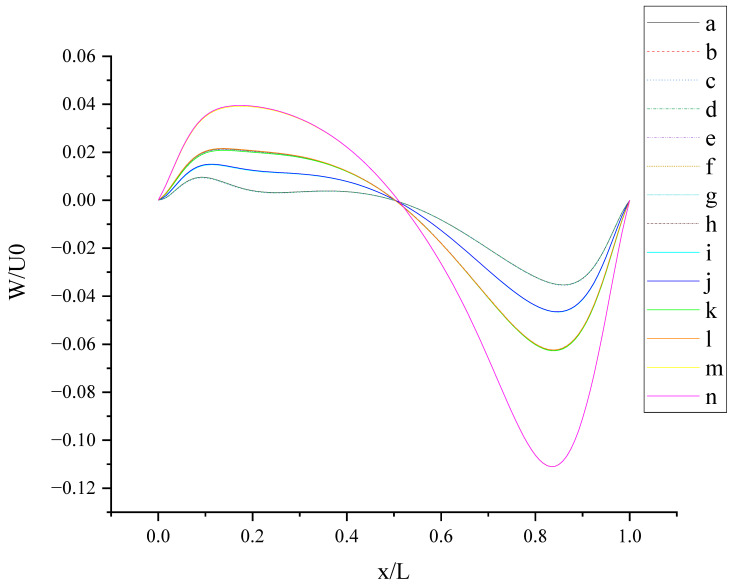
W along the centerline *X*-axis at t = 0.25 T.

**Figure 21:**
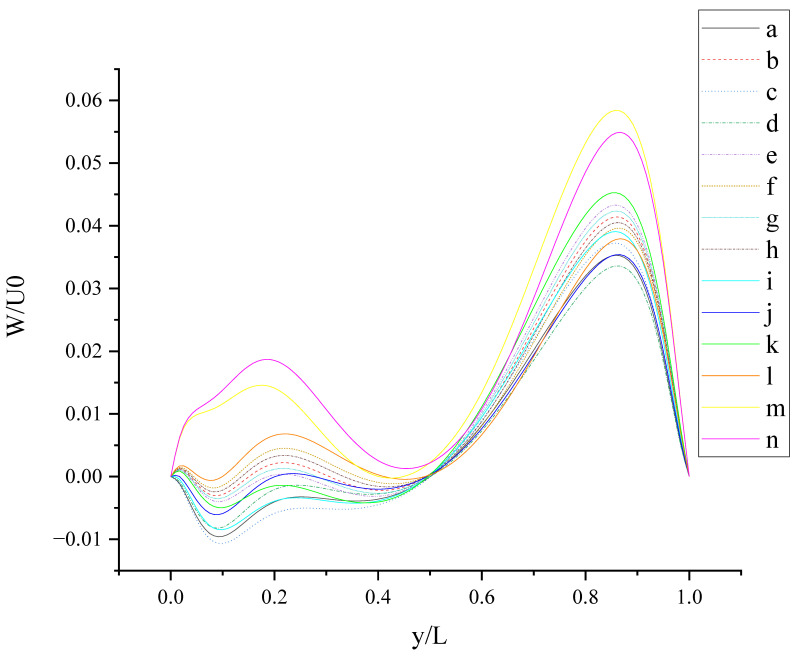
W along the centerline *Y*-axis at t = 0.25 T.

**Figure 22:**
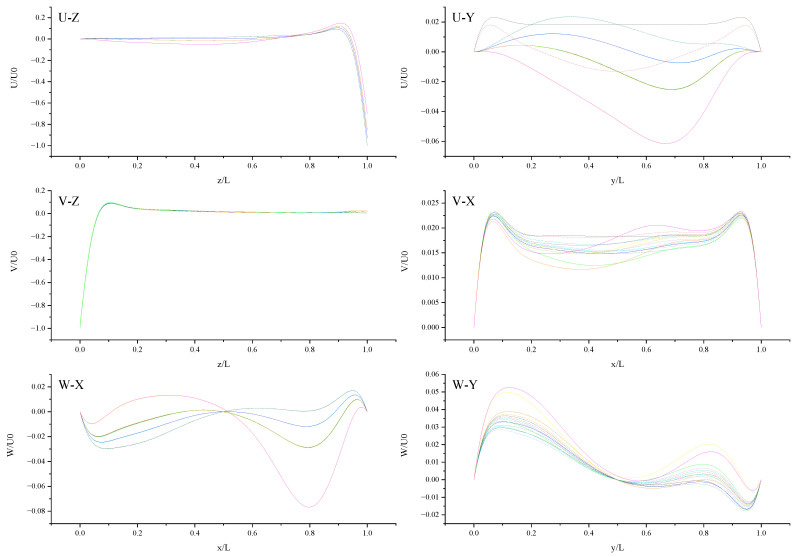
Centerline velocity profiles in X, Y, and Z directions at t = 0.5 T.

The contours for velocity U, V, and W and the vorticity magnitude of cases (a-n) at t = T, 0.25 T and 0.5 T are shown in Supplementary Material. It is found that nonphysical phenomena and numerical singularity do not exist, which shows that the present method is effective and the present results are credible. Furthermore, the centerline velocity profiles in the X, Y, and Z directions at t = T, 0.25 T and 0.5 T are shown in Figure 10, Figure 11, Figure 12, Figure 13, Figure 14, Figure 15, Figure 16, Figure 17, Figure 18, Figure 19, Figure 20, Figure 21 and Figure 22.

Figure 10 shows U (the velocity component in the X direction) along the centerline *Z*-axis at t = T and its magnified view. As shown in Figure 10, the 14 curves are divided into four groups according to the level of *b_x_* (*b_x_* = 0, 0.05, 0.1, and 0.2): group 1: cases (a, c, and d); group 2: cases (i and j); cases (b, e, f, g, h, k, and l); cases (m, and n). The slip length *b_x_* has greater influence on U than *b_y_*. It is found that velocity profiles in each group are very close. Therefore, for *b_x_* at the same level, the existence of *b_y_* on the top or bottom wall has almost no influence on the change in U along the centerline *Z*-axis. The greater *b_x_* is, the greater the influence it has on the change in U along the centerline *Z*-axis for z/L < 0.9 (U < 0). For z/L > 0.9, U increases rapidly, and the closer the location is to the top wall, the faster U increases, and the greater the velocity gradient. When the curves intersect, the relative magnitude of the curves changes. The distribution of the intersection points is chaotic, as shown in the red circle in Figure 10. Therefore, when *b_x_* = 0.1, the promotion effect of *b_y_* on increasing U will change with the change in of z.

Figure 11 shows U (the velocity component in the X direction) along the centerline *Y*-axis at t = T and its magnified view. As shown in Figure 11, U is negative along the centerline *Y*-axis in cases (c-n), which indicates that the existence of *b_y_* on the top wall or bottom wall results in the negative U along the centerline *Y*-axis. The anisotropic slip on the top wall with a larger slip length has a greater influence on the negative U along the centerline *Y*-axis, such as case (l) and case (n). For y/L < 0.52018, the absolute value of U in case (n) is less than that in case (l). For y/L > 0.52018, the absolute value of U in case (l) is less than that in case (n). Maybe there are more intersecting points near y/L = 0.5 and y/L = 0.915 because of the motion of the top and bottom walls and the interaction of the sidewalls and fluid.

Figure 12 shows V (the velocity component in the Y direction) along the centerline *Z*-axis at t = T and its magnified view. As shown in Figure 12, the results of different slip combinations are relatively close with a slight difference, indicating that anisotropic slip has a minor influence on V along the centerline *Z*-axis. However, it can still be seen that the slip combination in case (l) has the greatest influence on V along the centerline *Z*-axis, where the absolute value of negative V is the largest, as shown in the magnified view. Near the top wall, the anisotropic slip in case (l) results in the maximum positive V. For z/L > 0.06554, the intersection points of the curves are mostly evenly distributed along the centerline *Z*-axis, indicating that the strong or weak influence of slip distribution on V will change at most positions along the centerline *Z*-axis. It may be caused by the time-dependent motion of both top and bottom walls. Maybe, in this condition, the velocity V is mainly influenced by the disordered flow driven by the walls, and the influence of slip on V is negligible. So, the effect of slip may be greatly reduced in the disordered flow.

Figure 13 shows V (the velocity component in the Y direction) along the centerline *X*-axis at t = T. As shown in Figure 13, all curves have two troughs. For the no-slip condition, two troughs are located at x/L ≈ 0.07 and x/L ≈ 0.93. The slip condition makes the troughs closer to the center than the no-slip condition. Under the action of anisotropic slip, two peaks appear in the curve. The fluctuation range is large at large slip lengths, such as case (l) and case (n). Compared with pure slip in both top and bottom walls, anisotropic slip on the top wall results in stronger fluctuation disturbance and increases the 3D mixing of flow. The results of different slip combinations are similar near the side walls, and a great difference occurs in the cavity (0.2 < x/L < 0.8). The influence of slip on flow hardly propagates to the side walls, but mostly to the cavity.

Figure 14 shows W (the velocity component in the Z direction) along the centerline *X*-axis at t = T and its magnified view. As shown in Figure 14, 14 curves were divided into four groups according to the level of *b_x_*: *b_x_*= 0,0.05, 0.1, 0.2. The larger *b_x_* is, the greater the influence it has on the change in W along the centerline *X*-axis, and the larger the fluctuation range is, the closer the position of the peak or trough is to the center. For the same level of *b_x_*, the existence of *b_y_* on the top or bottom wall has little effect on the change in W along the centerline *X*-axis. For x/L ≈ 0.5, the direction of W reverses under the interaction of the top and bottom walls. This can be explained by the fact that the liquid inside the cavity flows clockwise.

Figure 15 shows W (the velocity component in the Z direction) along the centerline *Y*-axis at t = T. As shown in Figure 15, the large slip length will enhance the oscillation phenomenon of W along the centerline *Y*-axis and promote the increase in fluctuation amplitude, such as case (m) and case (n). This can be explained by the fact that the large slip length significantly increases the moving velocity of the wall. The existence of *b_y_* on the top wall has a stronger effect on enhancing the amplitude of the left peak, and the existence of *b_y_* on the bottom wall has a stronger effect on enhancing the amplitude of the right peak. The direction of *b_y_* intersects with the motion direction of the top wall vertically, enhancing the disturbance of W near the left side wall; the direction of *b_y_* is the same as the motion direction of the bottom wall, enhancing the disturbance of W near the right sidewall. So, the influence of slip on flow also depends on the slip direction and wall motion direction.

Figure 16 shows U (the velocity component in the X direction) along the centerline *Z*-axis at t = 0.25 T and its magnified view. As shown in Figure 16, the 14 curves are divided into four groups according to the level of *b_x_* (*b_x_* = 0, 0.05, 0.1, 0.2): group 1: cases (d, a, and c); group 2: cases (j and i); cases (l, f, h, b, g, e, and k); cases (n and m). It is found that velocity profiles for U in each group are very close. Therefore, for *b_x_* at the same level, the existence of *b_y_* on the top or bottom wall has almost no influence on the change in U along the centerline *Z*-axis. So, the direction of slip is also an important consideration. The larger *b_x_* results in a larger peak near the top wall, which has a greater influence on the change in U along the centerline *Z*-axis. When *b_x_* and *b_y_* are fixed, the anisotropic slip on the top wall has a greater effect on the positive U than the pure slip on the top and bottom walls. This may be explained by the directional inconsistency. When U > 0, there is no intersection point in 14 curves, and with fixed *b_x_* = 1, larger *b_y_* on the top wall results in larger U. With the existence of *b_y_* on the top wall, the increase in velocity is facilitated.

Figure 17 shows U (the velocity component in the X direction) along the centerline *Y*-axis at t = 0.25 T. As shown in Figure 17, the pure slip on the top and bottom walls makes the curve symmetrical, and the anisotropic slip on the top wall makes the trough closer to the right-side wall. The asymmetry can be caused by the direction of slip normal to the wall motion direction.

Figure 18 shows V (the velocity component in the Y direction) along the centerline *Z*-axis at t = 0.25 T and its magnified view. With the existence of *b_y_*, the slip velocity component in the Y direction appears on the top wall, and the larger slip length makes the non-negative value of V larger, such as with the case (l). In the magnified view, the order of peak value is k, c, i, e, m, g, a, d, b, j, h, f, l, and n. With the existence of *b_y_* on the bottom wall, the larger *b_y_* results in a larger peak value. The existence of *b_y_* can contribute to influencing the flow. With fixed *b_y_* = 0.1, the larger *b_x_* results in a smaller peak value.

At t = 0.25 T, the top and bottom walls move with oscillating velocity U = U_0_cos(*ωt*) = 0, but the slip drives the flow in the cavity. The trough near the bottom wall is more obvious in Figure 19 than that in Figure 13, owing to the different oscillating velocity. The trend in curves in Figure 20 is similar to that in Figure 14. The order of peak value near the right side wall in Figure 21 is consistent with that in Figure 15, which shows that the oscillating velocity of the top and bottom wall has a weaker influence on W than on U and V. It is found that W is positive along the centerline *Y*-axis in case (n) at t = 0.25 T. Anisotropic slip with large slip length can result in the disruptive change. Maybe, in this condition, the flow is dominated by anisotropic slip.

As shown in Figure 22, the profiles of U along the centerline *Y*-axis at t = 0.5 T show no qualitative similarity with those at t = T and t = 0.25 T. The other five types of profiles at t = 0.5 T show some approximative mirror symmetry with those at t = T. Because the top and bottom walls move with oscillating velocity U = U_0_cos(*ωt*), the direction of velocity at t = 0.5 t is opposite to that at t = T.

As shown in Figures S1, S5, S6, S10, S11 and S15 in the Supplementary Materials, the contour of vorticity magnitude is concentrated on the top and bottom walls, owing to shear stress affected by the motion of the top and bottom walls. As shown in Table 2, the maximum vorticity magnitude at t = T and 0.5 T is about an order of magnitude larger than that at t = 0.25 T, owing to the time-dependent oscillating velocity of the top and bottom walls. Compared to the no-slip case, the maximum vorticity magnitude in slip cases changes very little at t = T and 0.5 T, and the maximum percentage change is 3% in case (k). Compared to the no-slip case, case (m) and case (n) obtain about a 120% increase in the percentage of the maximum vorticity magnitude at t = 0.25 T. It is found that the maximum vorticity magnitude makes no change at t = T and 0.5 T when the anisotropic slip exists on the top wall.

## 5. Conclusions

The present method is validated by simulating the microchannel flow in 3D. Compared with the reference data, the present method is more accurate than the bounce-back and specular reflection slip boundary condition in LBM in Ref. [11]. The effect of anisotropic slip boundary conditions on turbulent flow is investigated by considering different slip lengths in streamwise and spanwise directions. Good agreement with DNS results shows that the present method is also accurate and stable to simulate fluid slip on 3D hydrophobic microchannel walls in a turbulent flow. The present method is effectively accurate and stable to capture velocity profiles and predict drag changes to study the effect of anisotropic slip. Then, the present method is applied to the two-sided, orthogonal, oscillating, micro-lid-driven cavity flow. Some findings are obtained from the simulation results, which can help in better understanding the anisotropic slip effect on the unsteady microflow and the design of microdevices:

The oscillating velocity of the wall has a weaker influence on W than on U and V. In most cases, large slip length has a more significant influence on velocity profiles than small slip length. However, for V along the centerline *Z*-axis at t = 0.25 T, the larger streamwise slip length on the top wall results in a smaller peak value with a fixed spanwise slip length. Compared with pure slip in both top and bottom walls, anisotropic slip on the top wall has a greater influence on flow, increasing the 3D mixing of flow. In short, the influence of slip on the flow field depends not only on slip length but also on the relative direction of the wall motion and the slip velocity.

## Figures and Tables

**Table 1:** The velocity set for the D3Q19 lattice model.

$\mathrm{Velocities}\;\xi_{\alpha }$	Number	$\mathrm{Length}\;|\xi_{\alpha }|$	$\mathrm{Weight}\;W_{\alpha }$
(0,0,0)*c*	1	0	1/3
(±1, 0, 0)*c*, (0, ±1, 0)*c*, (0, 0, ±1)*c*	6	1	1/18
(±1, ±1, 0)*c*, (±1, 0, ±1)*c*, (0, ±1, ±1)*c*	12	$\sqrt{2}$	1/36

**Table 2:** Maximum vorticity magnitude at t = T, 0.25 T, 0.5 T.

	t	a	b	c	d	e	f	g	h	i	j	k	l	m	n
Case															
T		11.3139	11.3139	11.4836	11.3139	11.4836	11.3139	11.3988	11.3139	11.4836	11.3139	11.6533	11.3139	11.4836	11.3139
0.25 T		1.08977	1.20004	1.0898	1.09042	1.20004	1.20603	1.20004	1.20154	1.07988	1.08963	1.20004	1.22381	2.40009	2.40309
0.5 T		11.3139	11.3139	11.1442	11.3139	11.1442	11.3139	11.2291	11.3139	11.1442	11.3139	10.9745	11.3139	11.1442	11.3139

## Data Availability

The data that support the findings of this study are available within the article.

## Supplementary Materials

The following supporting information can be downloaded at: https://www.mdpi.com/article/10.3390/e24070907/s1. Figure S1: Contours for Velocity U, V, W and Vorticity magnitude at t = T in case (a) and case (b). Figure S2: Contours for Velocity U at t = T in cases (c–n). Figure S3: Contours for Velocity V at t = T in cases (c–n). Figure S4: Contours for Velocity W at t = T in cases (c–n). Figure S5: Contours for Vorticity magnitude at t = T in cases (c–n). Figure S6: Contours for Velocity U, V, W and Vorticity magnitude at t = 0.25 T in case (a) and case (b). Figure S7: Contours for Velocity U at t = 0.25 T in cases (c–n). Figure S8: Contours for Velocity V at t = 0.25 T in cases (c–n). Figure S9: Contours for Velocity W at t = 0.25 T in cases (c–n). Figure S10: Contours for Vorticity magnitude at t = 0.25 T in cases (c–n). Figure S11: Con-tours for Velocity U, V, W and Vorticity magnitude at t = 0.5 T in case (a) and case (b). Figure S12: Contours for Veloc-ity U at t = 0.5 T in cases (c–n). Figure S13: Contours for Velocity V at t = 0.5 T in cases (c–n). Figure S14: Contours for Velocity W at t = 0.5 T in cases (c–n). Figure S15: Contours for Vorticity magnitude at t = 0.5 T in cases (c–n). Figure S16: Contours for Velocity U, V, W and Vorticity magnitude at t = 0.75 T in case (a) and case (b). Figure S17: Contours for Velocity U at t = 0.75 T in cases (c–n). Figure S18: Contours for Velocity V at t = 0.75 T in cases (c–n). Figure S19: Contours for Velocity W at t = 0.75 T in cases (c–n). Figure S20: Contours for Vorticity magnitude at t = 0.75 T in cases (c–n).
