# Supplementary material for: Three-Dimensional Simulations of Anisotropic Slip Microflows Using the Discrete Unified Gas Kinetic Scheme
Source: Entropy (Basel). 2022 Jun 30;24(7):907. doi: 10.3390/e24070907 (PMC9316686; doi:10.3390/e24070907)
Supplement: Supplementary file 1 [file entropy-24-00907-s001.zip › entropy-1764001-supplementary.pdf]

Supplementary material. Contours for three velocity components and vorticity magnitude

In the supplementary material, the contours for three velocity components and vorticity magnitude of cases (a-n) at t=T, 0.25T and 0.5T are present in the 3D view.

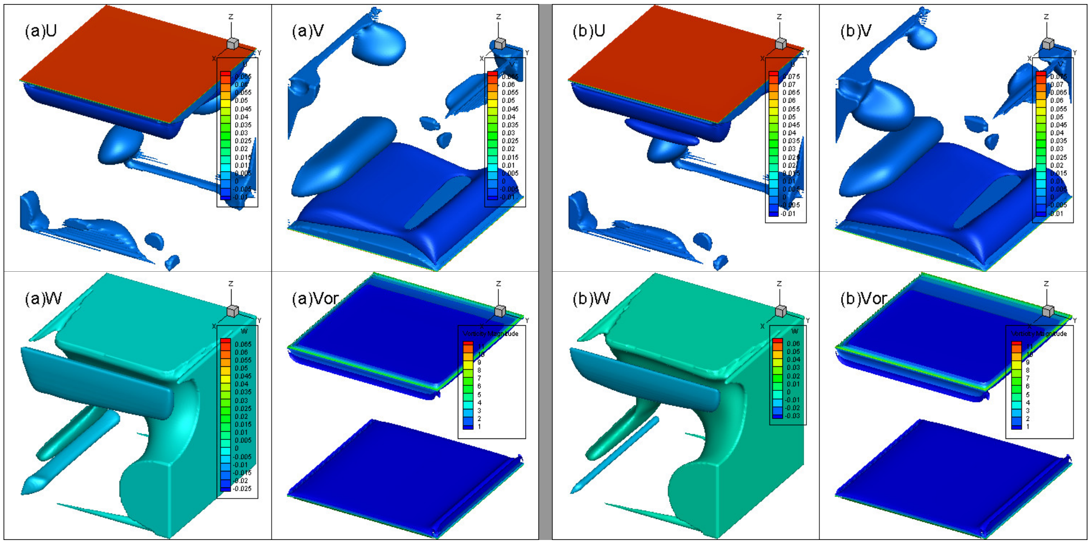

Figure S1. Contours for Velocity U, V, W and Vorticity magnitude at t=T in case (a) and case (b).

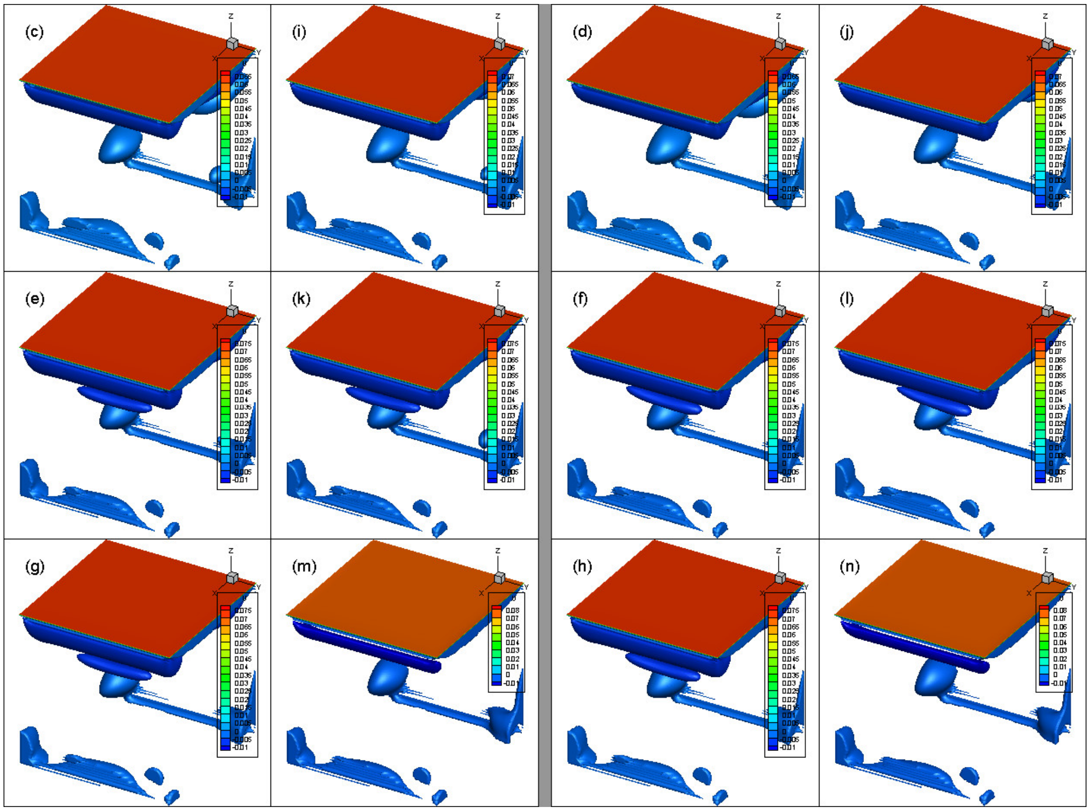

Figure S2. Contours for Velocity U at t=T in cases (c-n).

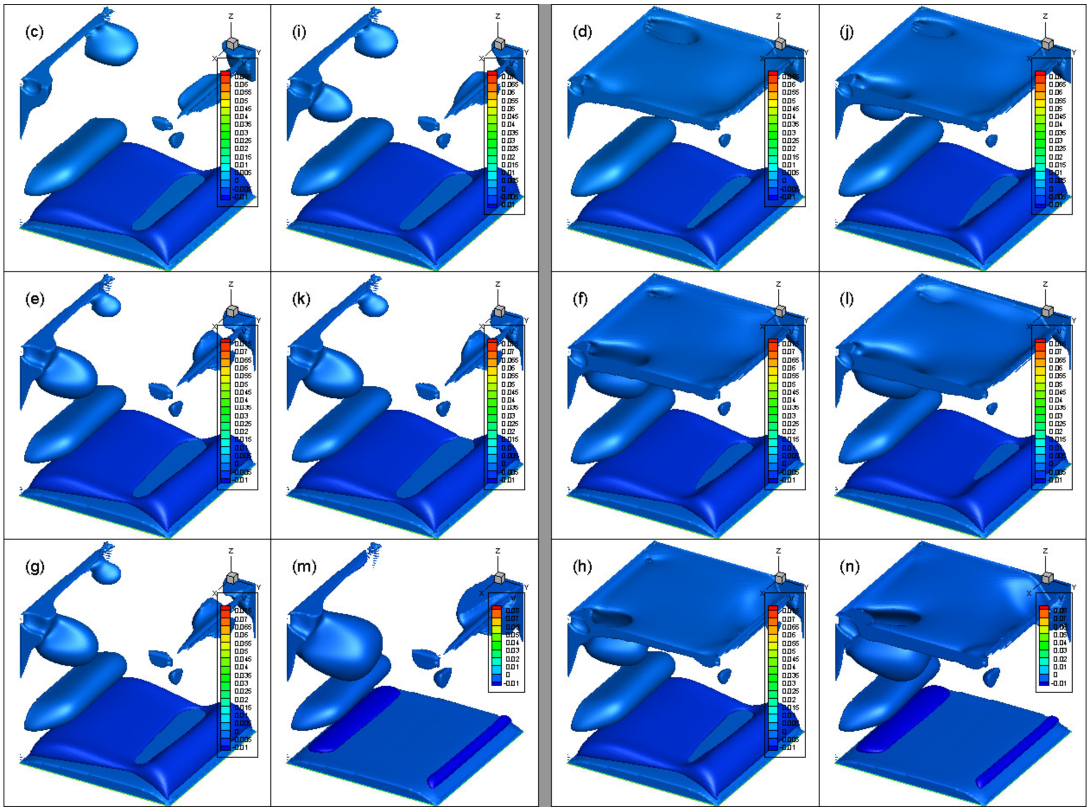

Figure S3. Contours for Velocity V at t=T in cases (c-n).

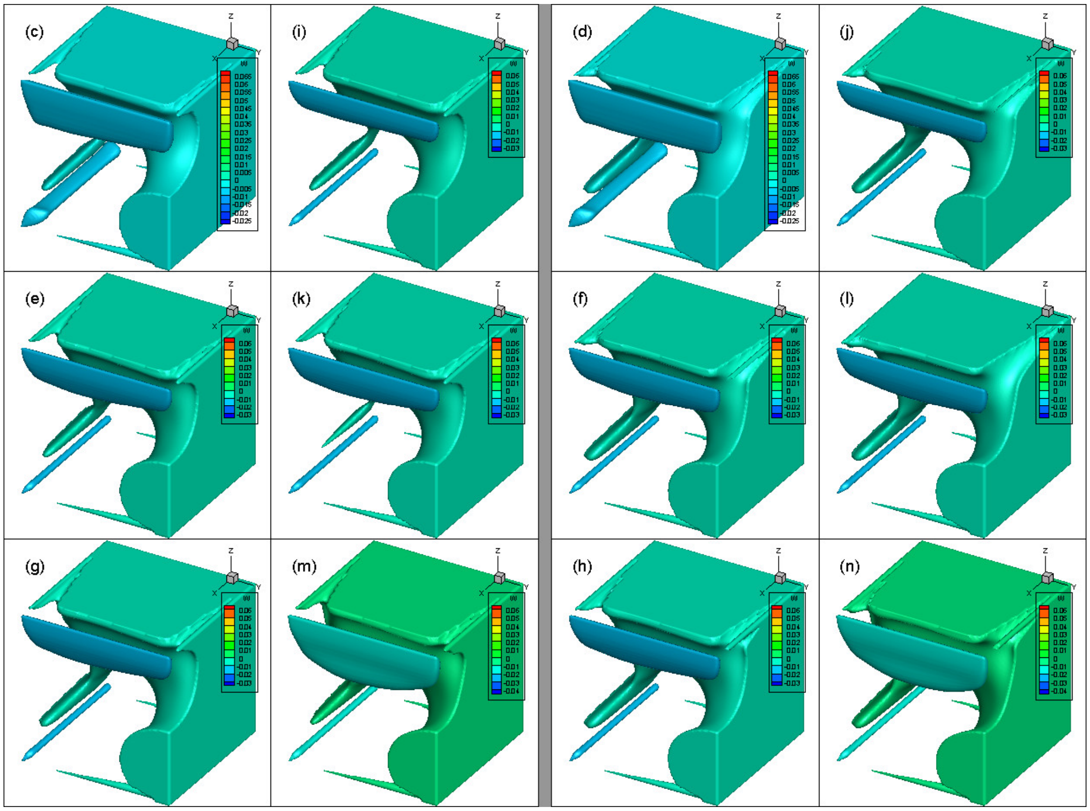

Figure S4. Contours for Velocity W at t=T in cases (**c**–**n**).

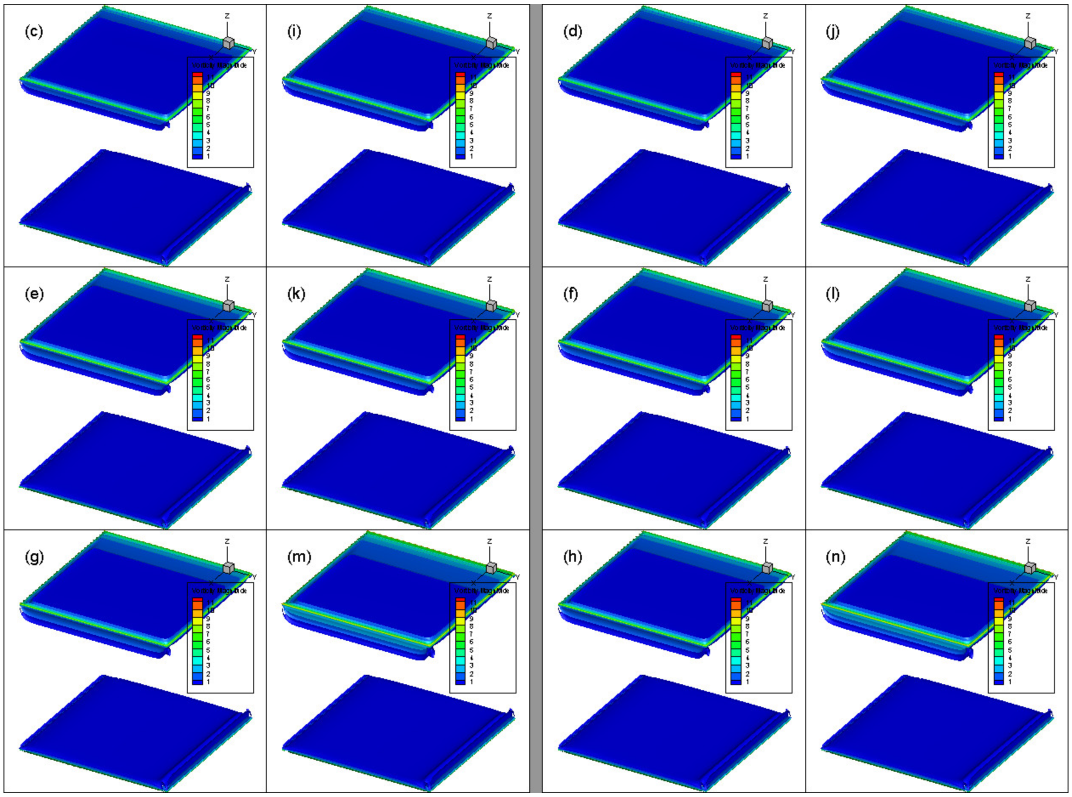

Figure S5. Contours for Vorticity magnitude at t=T in cases (**c**–**n**).

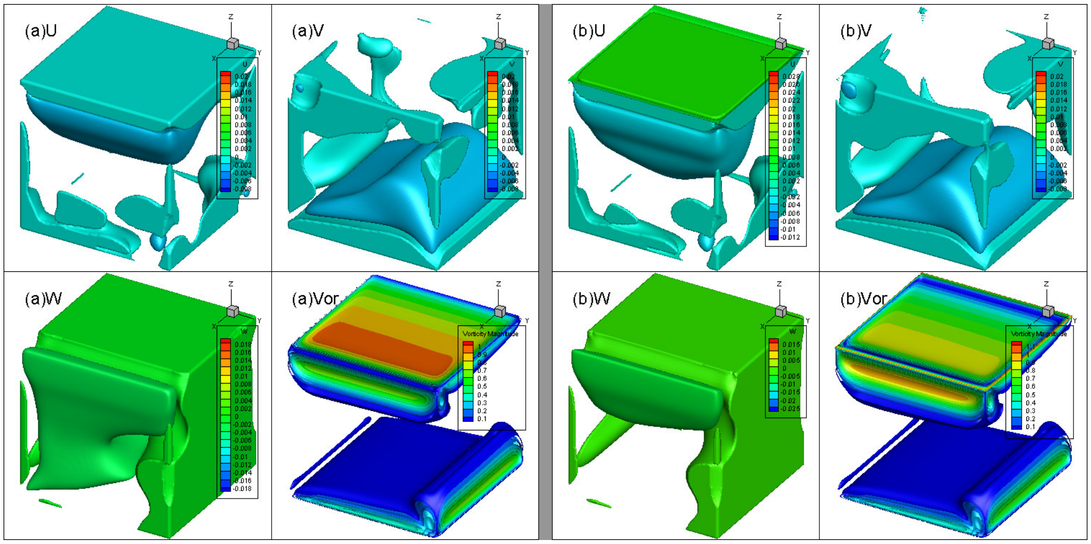

Figure S6. Contours for Velocity U, V, W and Vorticity magnitude at t=0.25T in case (**a**) and case (**b**).

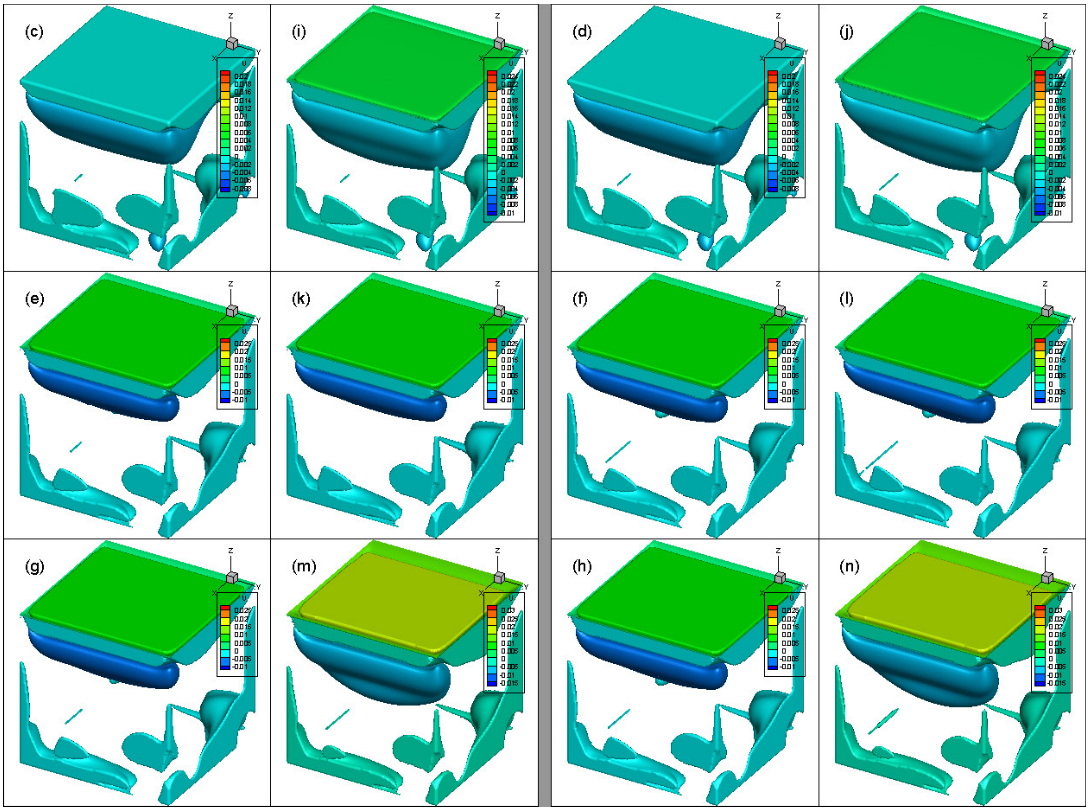

Figure S7. Contours for Velocity U at t=0.25T in cases (**c**–**n**).

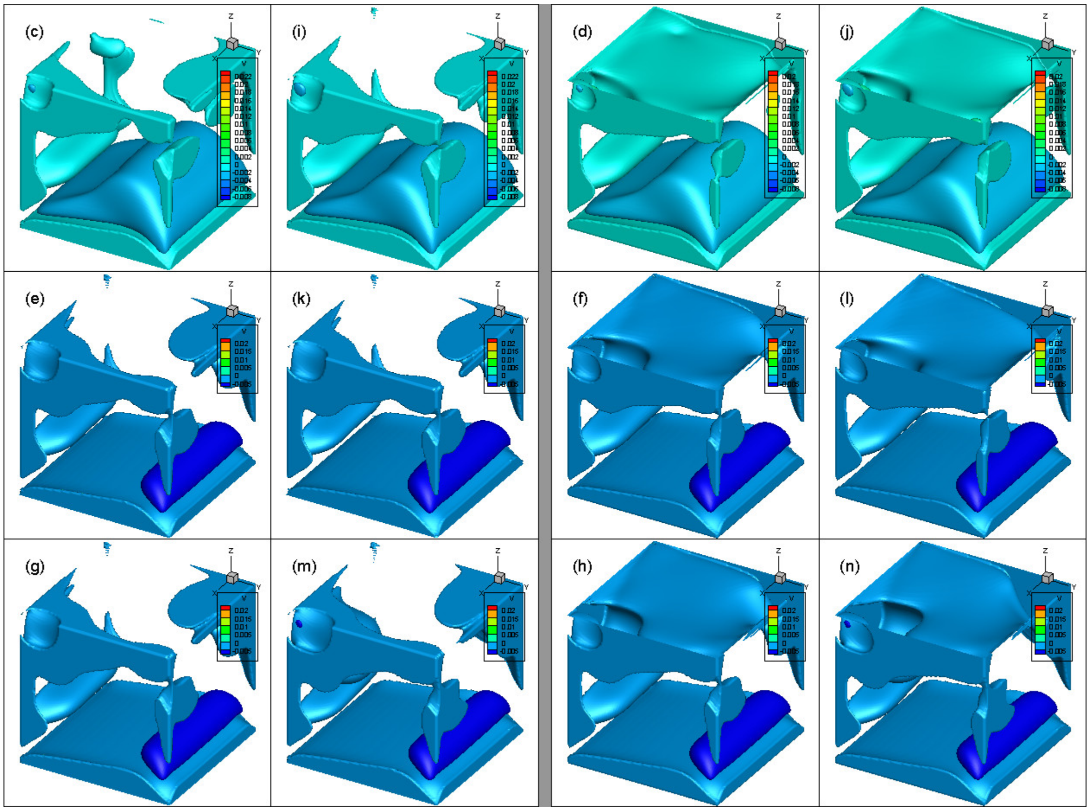

Figure S8. Contours for Velocity V at t=0.25T in cases (**c**–**n**).

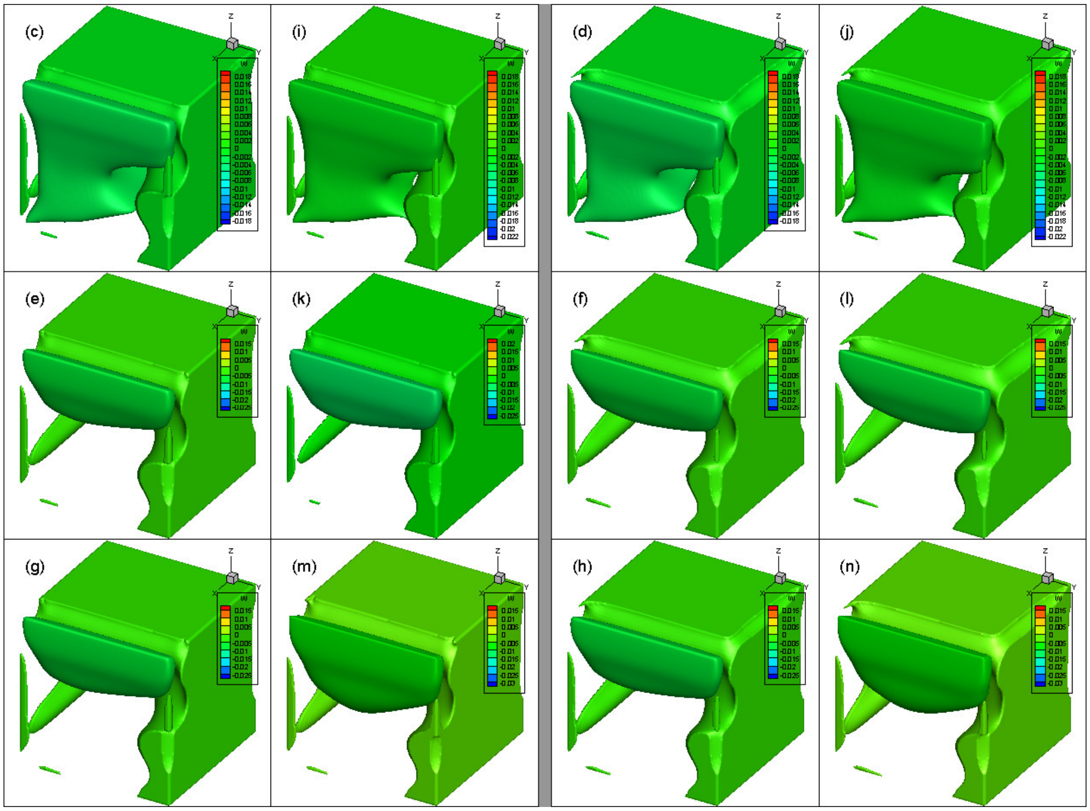

Figure S9. Contours for Velocity W at t=0.25T in cases (**c**-**n**).

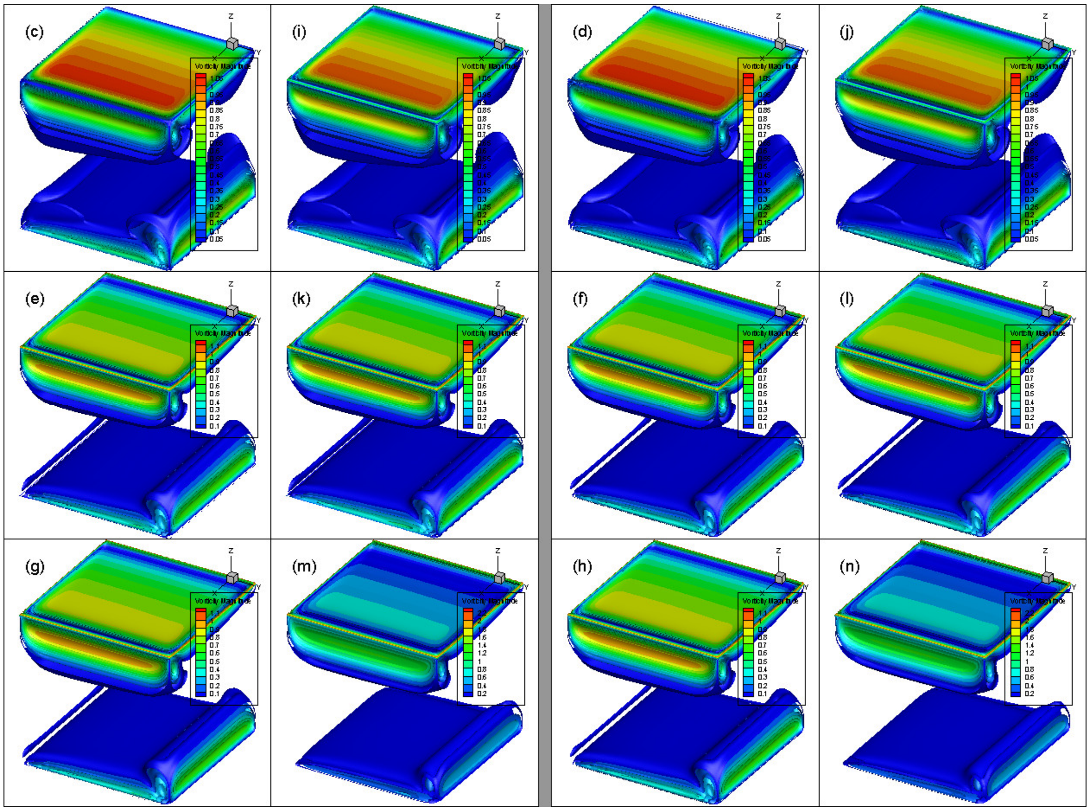

Figure S10. Contours for Vorticity magnitude at t=0.25T in cases (c-n).

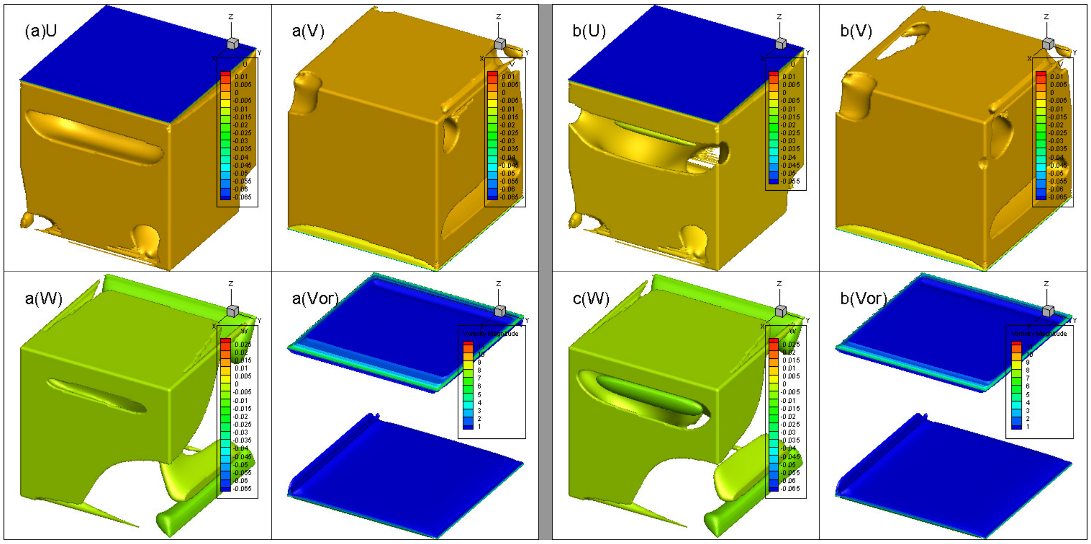

Figure S11. Contours for Velocity U, V, W and Vorticity magnitude at t=0.5T in case (**a**) and case (**b**).

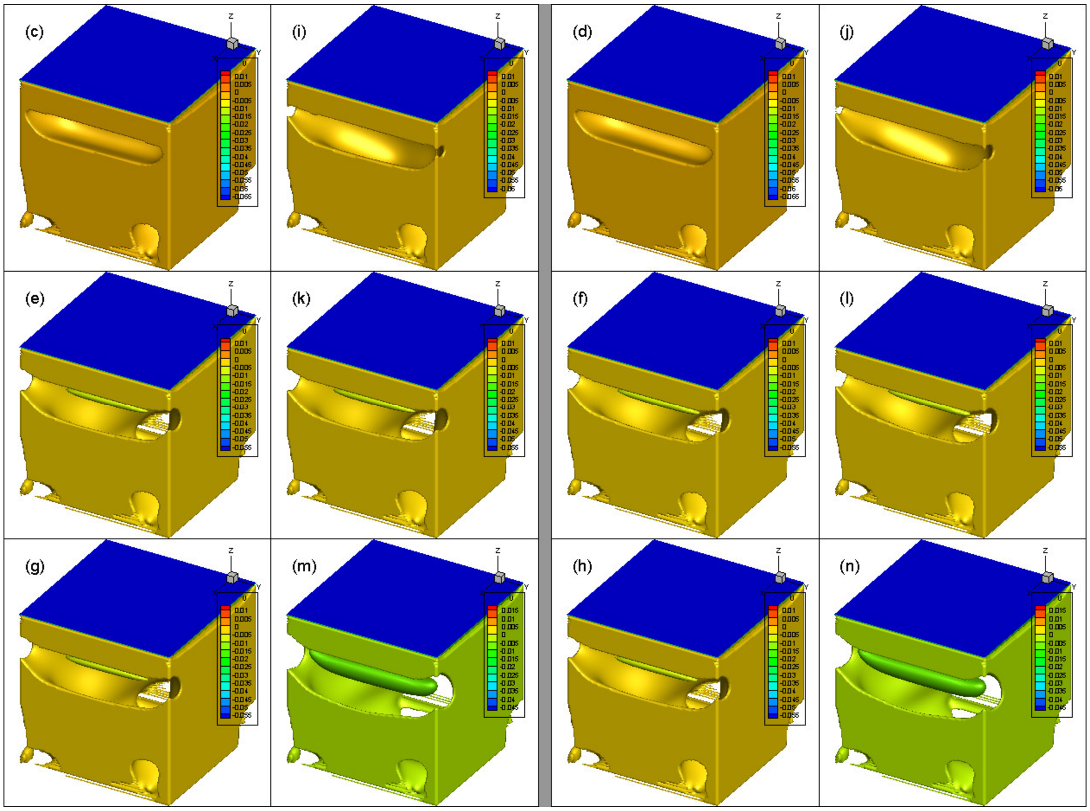

Figure S12. Contours for Velocity U at t=0.5T in cases (c-n).

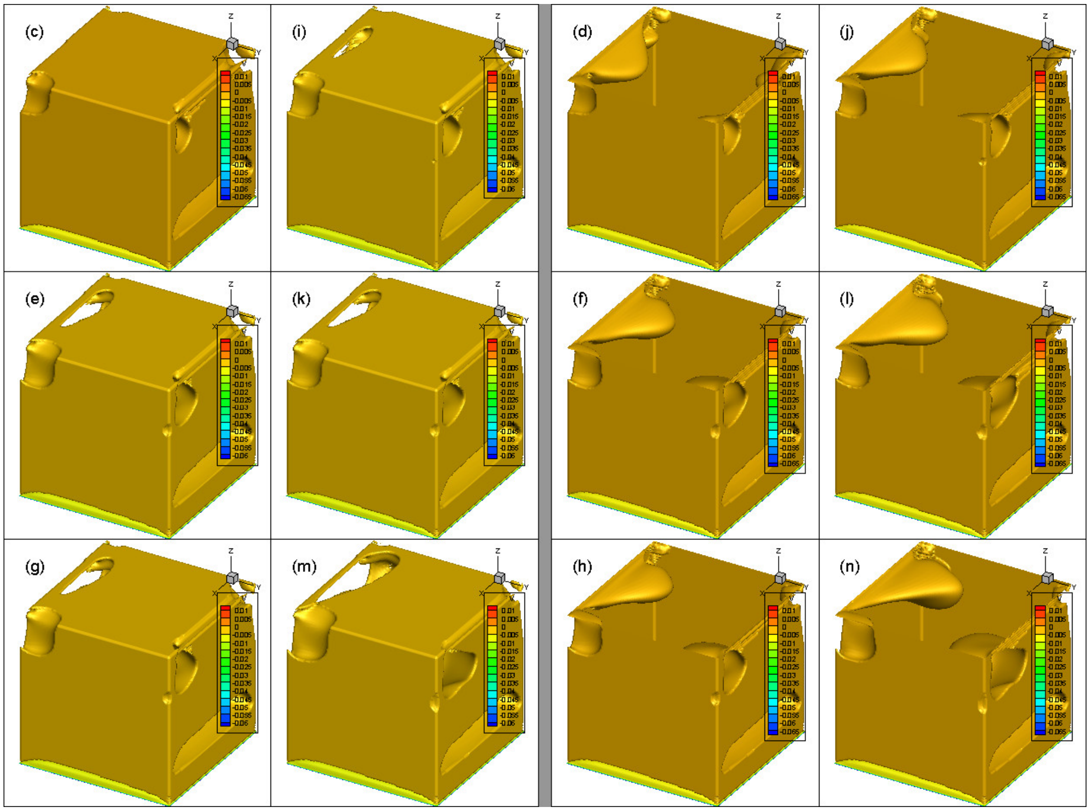

Figure S13. Contours for Velocity V at t=0.5T in cases (c-n).

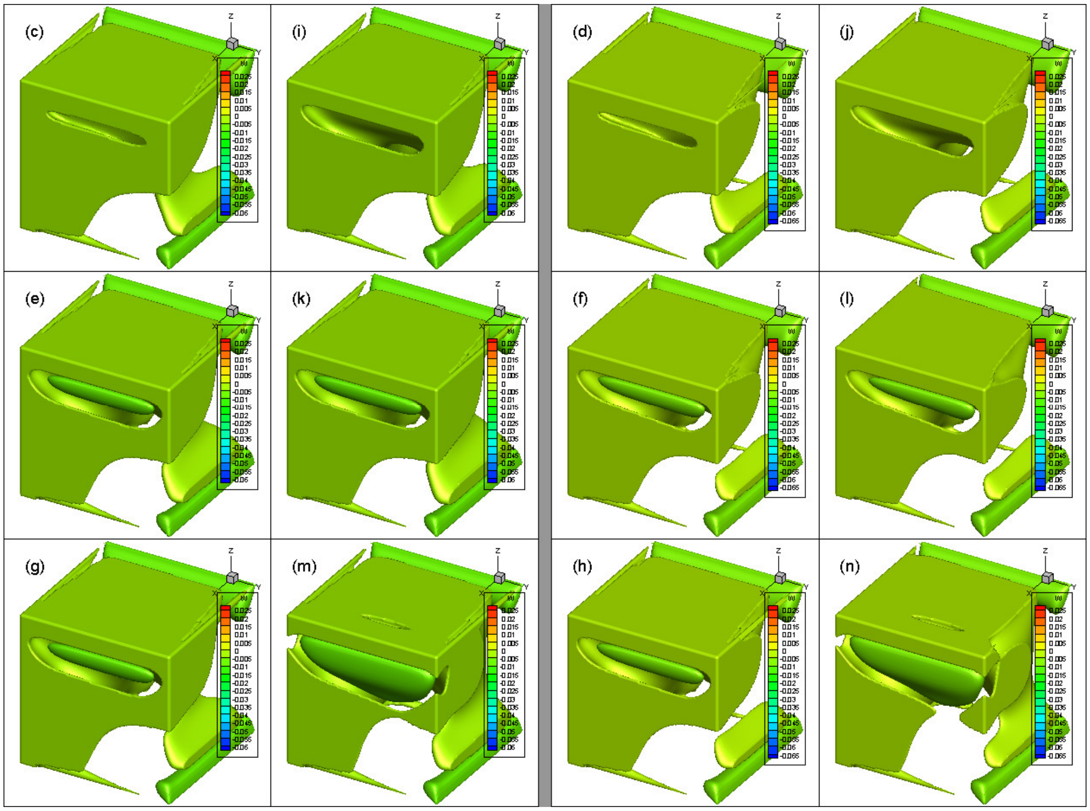

Figure S14. Contours for Velocity W at t=0.5T in cases (c-n).

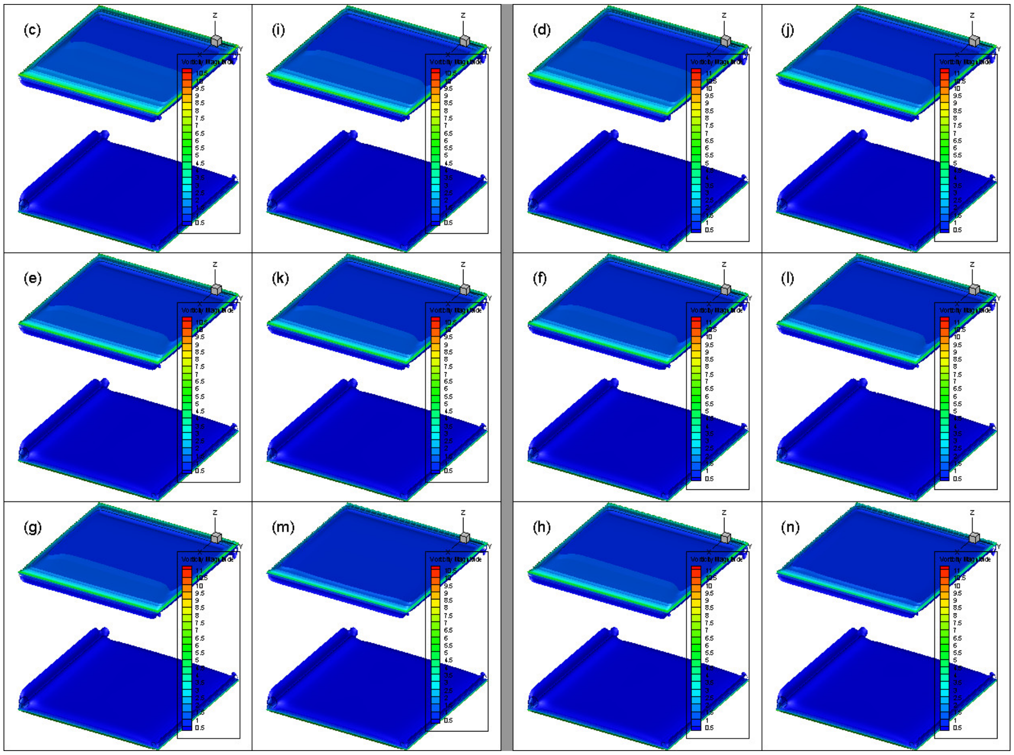

Figure S15. Contours for Vorticity magnitude at t=0.5T in cases (c-n).

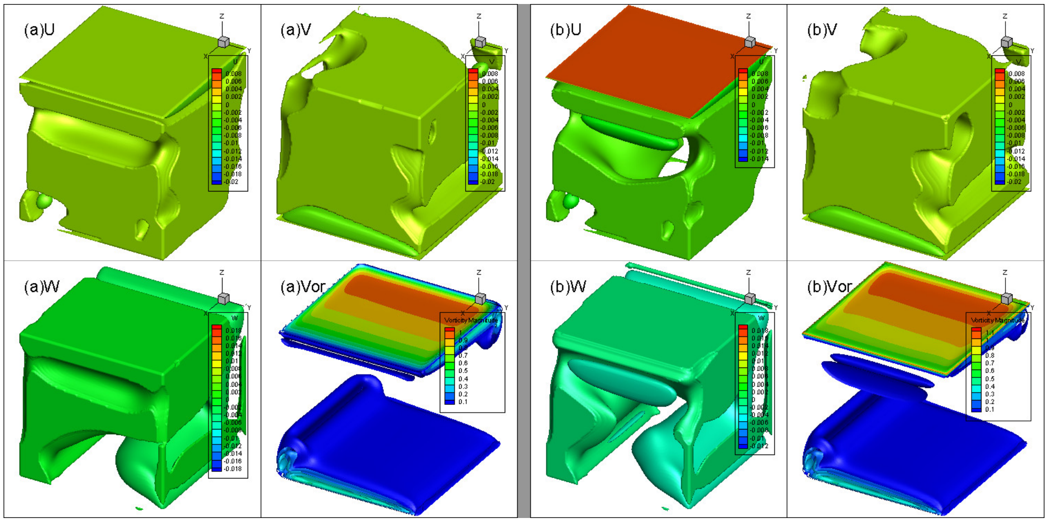

Figure S16. Contours for Velocity U, V, W and Vorticity magnitude at t=0.75T in case (a) and case (b).

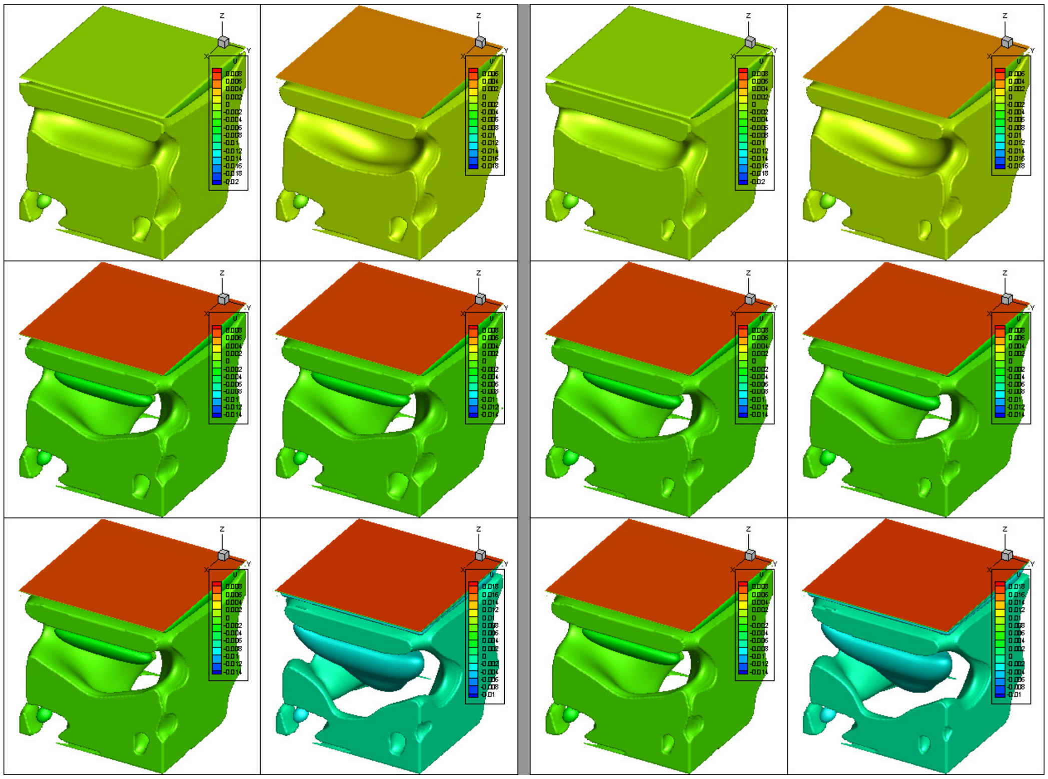

Figure S17. Contours for Velocity U at t=0.75T in cases (c-n).

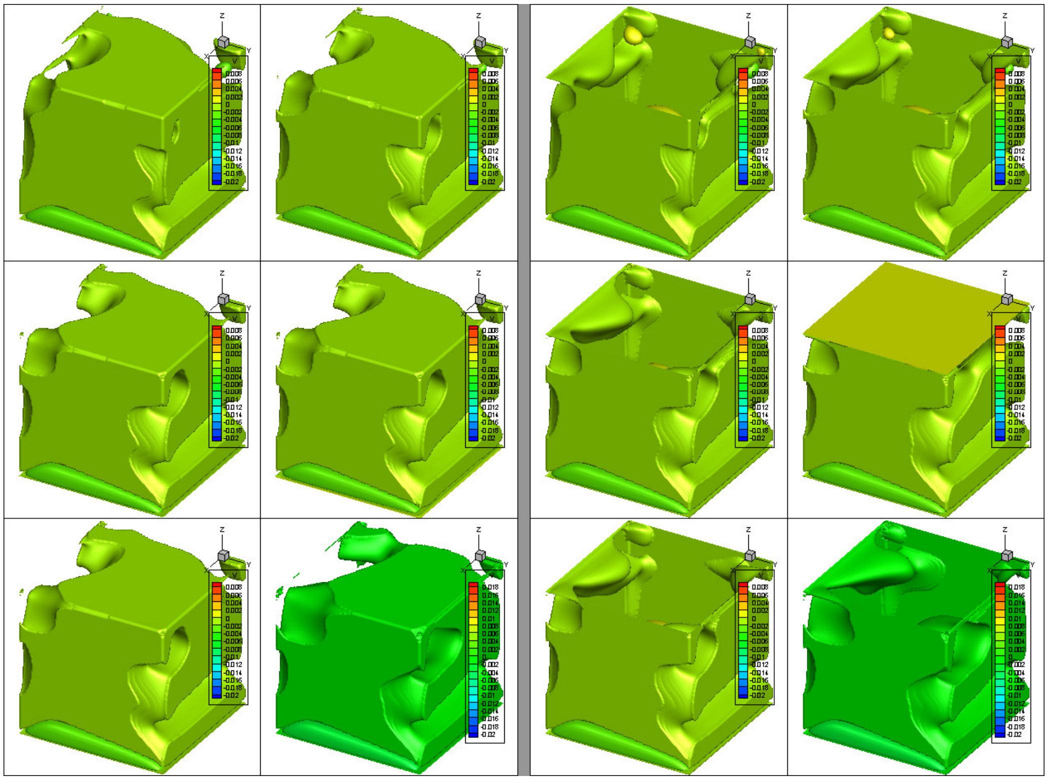

Figure S18. Contours for Velocity V at t=0.75T in cases (c-n).

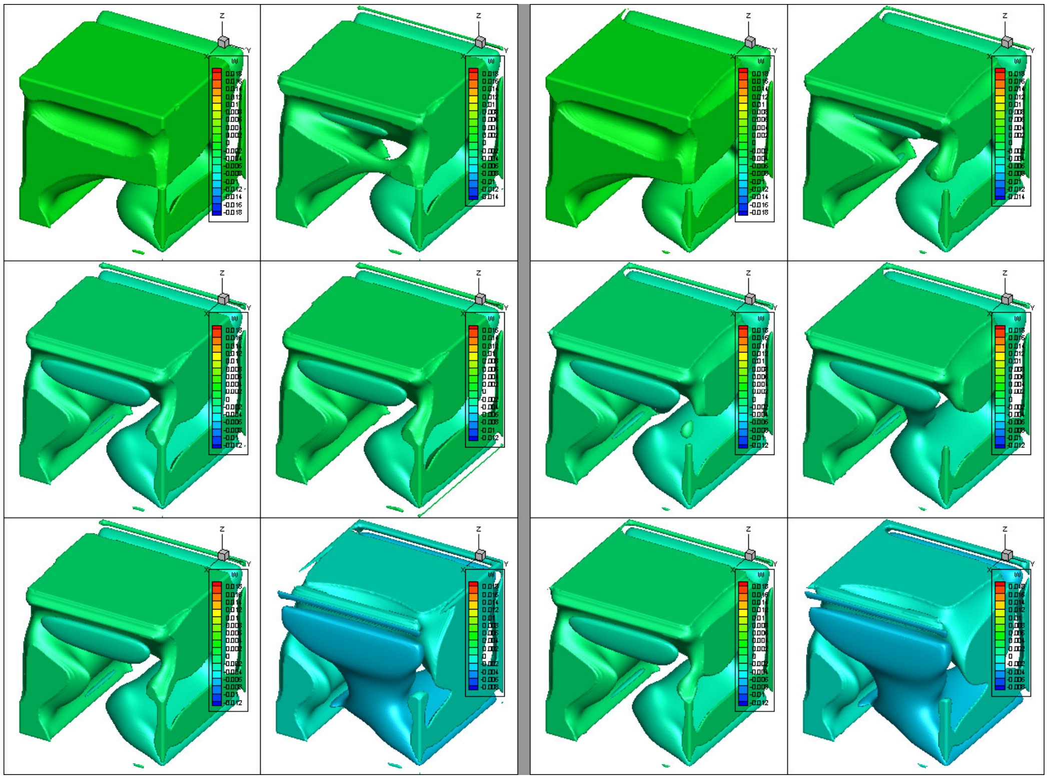

Figure S19. Contours for Velocity W at t=0.75T in cases (c-n).

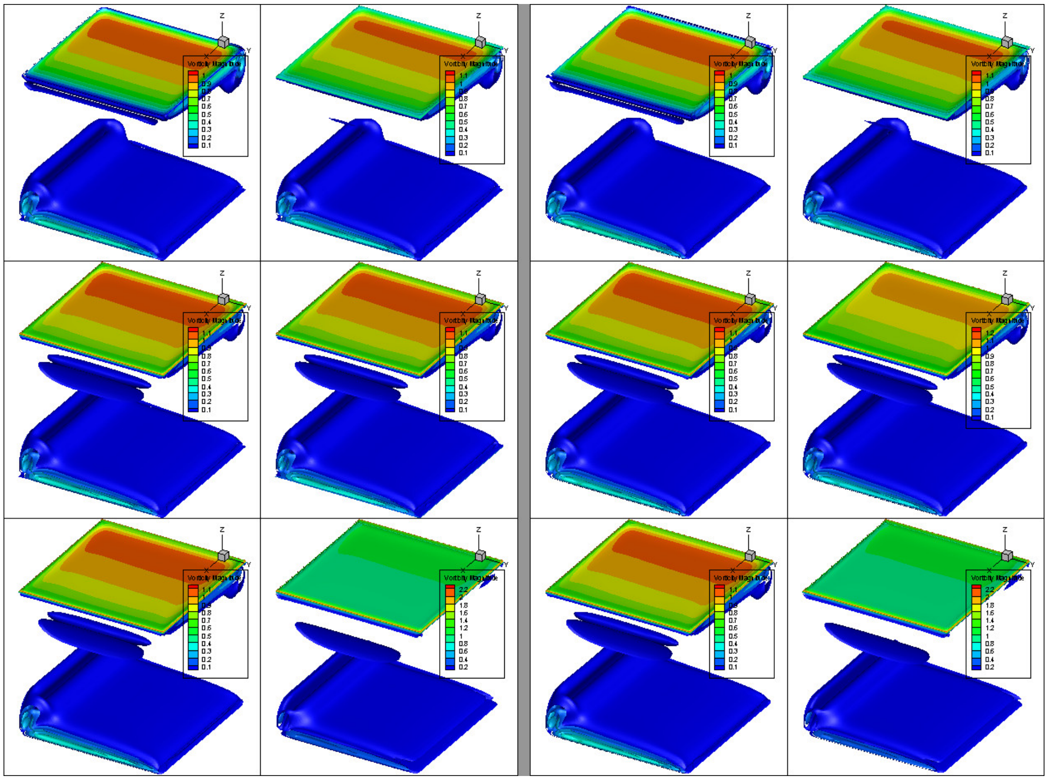

Figure S20. Contours for Vorticity magnitude at t=0.75T in cases (c-n).
